# Transcriptomic and proteomic host response to *Aspergillus fumigatus* conidia in an air-liquid interface model of human bronchial epithelium

**DOI:** 10.1371/journal.pone.0209652

**Published:** 2018-12-27

**Authors:** Amreen Toor, Luka Culibrk, Gurpreet K. Singhera, Kyung-Mee Moon, Anna Prudova, Leonard J. Foster, Margo M. Moore, Delbert R. Dorscheid, Scott J. Tebbutt

**Affiliations:** 1 Experimental Medicine, University of British Columbia, Vancouver, Canada; 2 Centre for Heart Lung Innovation, University of British Columbia and St. Paul’s Hospital, Vancouver, Canada; 3 Department of Biochemistry & Molecular Biology, University of British Columbia, Vancouver, Canada; 4 Department of Biological Sciences, Simon Fraser University, Burnaby, Canada; 5 Department of Medicine, Division of Respiratory Medicine, University of British Columbia, Vancouver, Canada; 6 Prevention of Organ Failure (PROOF) Centre of Excellence, Vancouver, Canada; Leibniz-Institut fur Naturstoff-Forschung und Infektionsbiologie eV Hans-Knoll-Institut, GERMANY

## Abstract

*Aspergillus fumigatus* (*A*. *fumigatus*) is a wide-spread fungus that is a potent allergen in hypersensitive individuals but also an opportunistic pathogen in immunocompromised patients. It reproduces asexually by releasing airborne conidiospores (conidia). Upon inhalation, fungal conidia are capable of reaching the airway epithelial cells (AECs) in bronchial and alveolar tissues. Previous studies have predominantly used submerged monolayer cultures for studying this host-pathogen interaction; however, these cultures do not recapitulate the mucocililary differentiation phenotype of the *in vivo* epithelium in the respiratory tract. Thus, the aim of this study was to use well-differentiated primary human bronchial epithelial cells (HBECs) grown at the air-liquid interface (ALI) to determine their transcriptomic and proteomic responses following interaction with *A*. *fumigatus* conidia. We visualized conidial interaction with HBECs using confocal laser scanning microscopy (CLSM), and applied NanoString nCounter and shotgun proteomics to assess gene expression changes in the human cells upon interaction with *A*. *fumigatus* conidia. Western blot analysis was used to assess the expression of top three differentially expressed proteins, CALR, SET and NUCB2. CLSM showed that, unlike submerged monolayer cultures, well-differentiated ALI cultures of primary HBECs were estimated to internalize less than 1% of bound conidia. Nevertheless, transcriptomic and proteomic analyses revealed numerous differentially expressed host genes; these were enriched for pathways including apoptosis/autophagy, translation, unfolded protein response and cell cycle (up-regulated); complement and coagulation pathways, iron homeostasis, nonsense mediated decay and rRNA binding (down-regulated). CALR and SET were confirmed to be up-regulated in ALI cultures of primary HBECs upon exposure to *A*. *fumigatus* via western blot analysis. Therefore, using transcriptomics and proteomics approaches, ALI models recapitulating the bronchial epithelial barrier in the conductive zone of the respiratory tract can provide novel insights to the molecular response of bronchial epithelial cells upon exposure to *A*. *fumigatus* conidia.

## Introduction

*A*. *fumigatus* is a saprotrophic fungus that is widely distributed in nature through the release of airborne conidiospores (conidia) [1,2]. These conidia, 2–3 μm in diameter, are ubiquitous in the atmosphere and most humans inhale several hundred conidia every day [1,3,4]. Upon inhalation, they are capable of reaching the terminal bronchioles and alveoli where they can bind to [5–7] or be internalized by airway epithelial cells (AECs) [8–10]. *A*. *fumigatus* is also an opportunistic pathogen and this interaction between conidia and AECs may result in the development of allergic, chronic or invasive aspergillosis in susceptible hosts [11,12]. The most common form of allergic aspergillosis is known as allergic bronchopulmonary aspergillosis (ABPA) and is characterized by a severe allergic reaction triggered by secretion of toxins and allergens from the developing fungus [11,13]. Susceptible hosts include individuals with allergic asthma or cystic fibrosis [14–16] and the global burden of patients with ABPA is estimated to exceed 4.8 million [17]. In patients with pre-existing pulmonary disease, e.g., chronic obstructive pulmonary disease, sarcoidosis or tuberculosis, exposure to *A*. *fumigatus* can cause chronic pulmonary aspergillosis (CPA) [18,19]. The most severe form of aspergillosis, called invasive aspergillosis (IA), affects immunocompromised individuals and is characterized by proliferation of fungal hyphae within pulmonary tissue that may spread to other organs via the bloodstream [20,21]. IA is estimated to occur in 5–25% of acute leukemia patients, 5–10% of patients who received an allogeneic bone marrow transplant, and in 0.5–5% of individuals after cytotoxic drug treatment of blood diseases or solid-organ transplantation [2,22]. Mortality rates range from 40 to 90% in high risk populations and depend on multiple factors such as host immune status, site of infection and treatment [23] and annual incidences reported globally for IA exceed 300,000 individuals [17].

The first step in colonization of the host is the interaction of conidia with AECs. Elucidating the molecular details of this interaction is important for understanding *A*. *fumigatus* pathogenesis. As previously stated, the host immune system plays an important role in determining which form of aspergillosis develops upon interaction of conidia with the airway epithelium, which serves as a primary point of contact and initiates the immune response [9]. Upon inhalation, *A*. *fumigatus* conidia can reach the lower respiratory tract (conducting zone) and penetrate deep into the distal respiratory tract (respiratory zone) as well [24]. The conducting zone consists of a pseudo-stratified, columnar bronchial epithelium, and includes mucus-secreting goblet cells, ciliated cells and basal cells [25]. In healthy individuals, inhaled conidia are eliminated by mucociliary clearance, a physical barrier generated by mucus-secreting goblet and ciliated cells in the bronchial epithelium. It traps inhaled conidia in the mucus and actively transports them by the beating of cilia towards the oropharyngeal junction to be either swallowed or expectorated [26]. However, in ABPA patients, inhaled conidia initiates a T_H_2-mediated inflammatory response resulting in inflammation, airway hyperresponsiveness, hyperplasia/metaplasia of mucus-secreting goblet cells and sub-epithelial fibrosis [27]. Human bronchial epithelial cell lines, such as BEAS-2B [28–31] and 16HBE14o- [8,32], have been used to model bronchial epithelial interactions such as those seen in ABPA patients. For example, Gomez et al. have previously shown that 16HBE14o- cells internalize 30–50% of bound *A*. *fumigatus* conidia within 6 hours of co-incubation, and genes associated with repair and inflammatory processes are differentially expressed upon exposure to *A*. *fumigatus* [8]. In contrast, in IA, inhaled conidia can enter the alveoli, germinate, and enter the bloodstream. Innate immune defenses that lead to recruitment of alveolar macrophages and neutrophils are usually lacking in immunocompromised individuals, resulting in fungal growth through the alveoli to the bloodstream [27]. The distal respiratory tract consists of alveolar epithelium with alveolar type I and type II cells, and alveolar macrophages [24]; hence, to model alveolar epithelial infection in IA, many studies have used a type II pneumocyte cell line derived from human lung carcinoma (A549) [33,34]. Unlike bronchial cell lines, A549 cells internalize 30% of bound conidia after 3 hours [10]. Transcriptional analyses of A549 cells using RNA-sequencing by Chen et al. have revealed up-regulated genes to be associated with immune response, chemotaxis and inflammatory response, and down-regulated genes to be associated with development, hemopoiesis and ion transport [34].

Previous studies from our laboratory and others have shown phagocytosis of *A*. *fumigatus* conidia *in vitro* by professional phagocytes and non-phagocytic cells grown in submerged cultures [8,9,35]. Majority of the studies conducted to model both ABPA and IA have used submerged monolayer cultures as *in vitro* infection models. However, conventional submerged monolayer cultures lack the anatomical barrier provided by the ciliary activity and mucus production, and therefore fail to recapitulate the mucocililary differentiation phenotype of the *in vivo* epithelium in the respiratory tract [36,37]. Bronchial epithelial cells can be grown at the air-liquid interface (ALI) for 21–28 days to generate polarized, pseudo-stratified epithelium that possesses tight junctions and contains basal cells, mucus-secreting goblet cells and ciliated cells [38,39]. For example, phagocytosis of *A*. *fumigatus* conidia by epithelial cells has been shown using 14-day old ALI cultures of human primary nasal epithelial cells [40] and porcine tracheal epithelial cells [41]. However, to our knowledge, there are no studies of the early molecular response of human airway cells to *A*. *fumigatus* conidia using well-differentiated ALI cultures.

To date, most studies of the interaction between host lung epithelial cells and *A*. *fumigatus* have measured conidial internalization [9,33,40,42,43], cytokine release [44–49] or activation of signaling proteins and pathways [29,42,50]. Fewer studies have analyzed the transcriptomic response of lung epithelial cells to conidia [8,34,50,51], and proteomic analyses of the conidia-epithelium interaction are limited; Fekkar et al., (2012) carried out a secretome analysis of human bronchial epithelial cells in response to *A*. *fumigatus* using differential in-gel electrophoresis.

High-throughput ‘omics’ analysis may provide a better understanding of the complex host response and reveal novel molecular mechanisms of the host-pathogen interaction [52]. Therefore, the aims of the current study were to carry out transcriptomic and proteomic analyses of the early molecular response of polarized bronchial epithelial cells to *A*. *fumigatus* conidia. To better recapitulate the *in vivo* bronchial epithelium, we used primary human bronchial epithelial cells (HBECs) grown for 21–28 days as ALI cultures that contained basal, mucus-secreting goblet and ciliated cells.

## Results

### Well-differentiated ALI cultures of primary HBECs show little evidence of conidia internalization at 6 hours post-exposure

**Fig 1:**
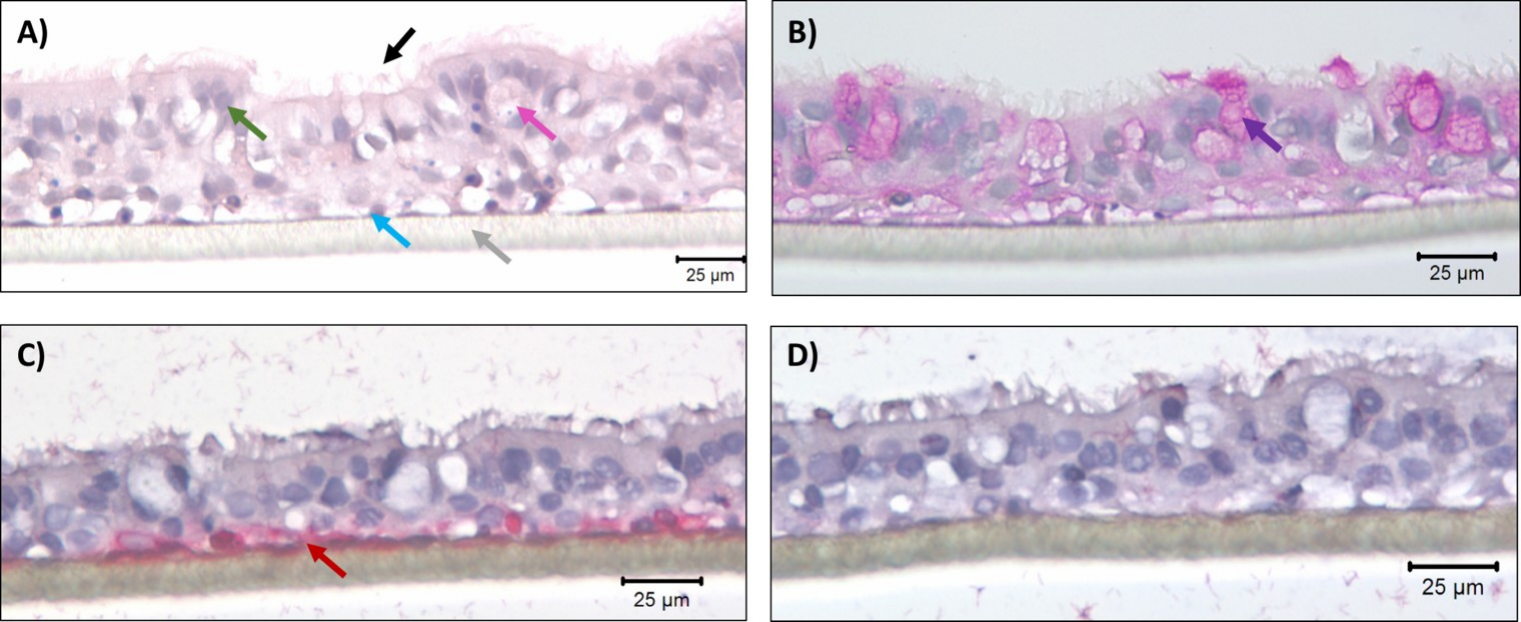
Representative images of primary HBECs as differentiated pseudostratified ALI cultures grown on 0.4 μm pore size polyethylene terephthalate (PET) membrane at 40X magnification. **(A)** Hematoxylin & Eosin (H&E) staining demonstrating pseudostratified epithelium with basal epithelial cells (blue arrow) on PET membrane (grey arrow). Additionally, mucus-secreting goblet cells (pink arrow) and ciliated cells (green arrow) are present. Black arrows indicate cilia on the apical side of the ALI culture. (B) Periodic acid-Schiff (PAS) stained ALI section showing mucus-secreting goblet cells (purple arrow). (C) ALI section demonstrates red stained Cytokeratin 5 (red arrow), a specific marker for basal epithelial cells. (D) ALI section showing blue nuclear counter staining only with mouse isotype control of the matched ALI section.

**Fig 2:**
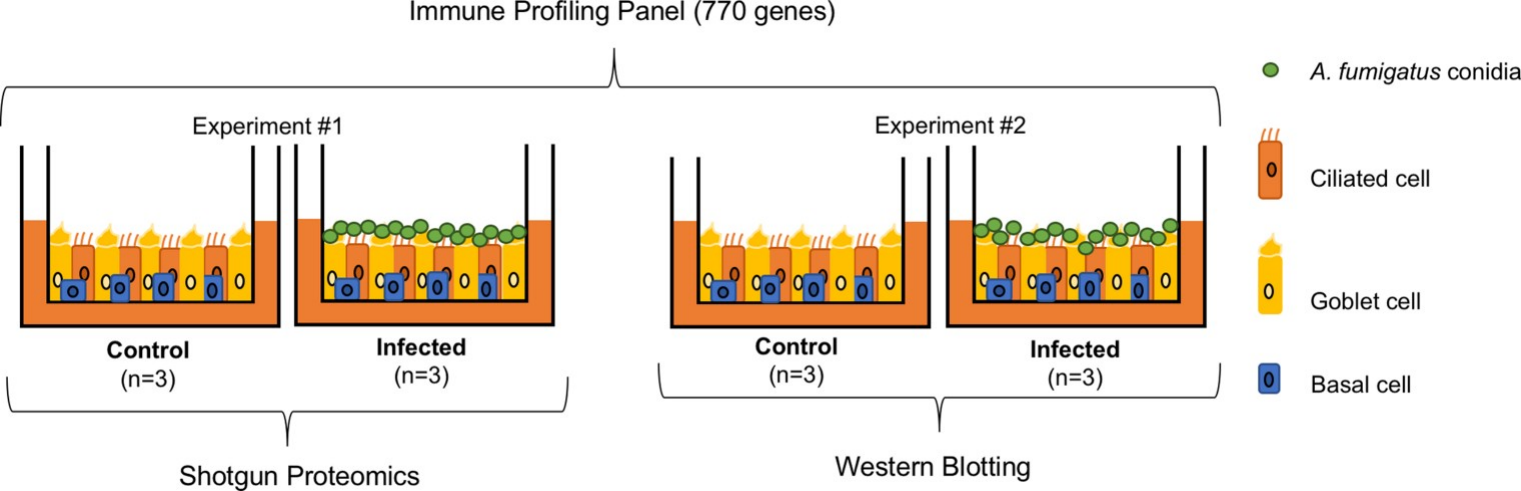
Overview of experimental design. Two independent experiments (experiment #1 and #2) were conducted. Each consisted of ALI cultures of primary HBECs incubated with PBS alone (control, n = 3) or *A*. *fumigatus* conidia suspended in PBS, MOI = 10 (infected, n = 3). Cells were exposed to conidia on the apical side for 6 hours at 37°C in both experiments. RNA from all 12 samples were assessed using NanoString’s Immune Profiling Panel. Proteins extracted from 6 samples in experiment #1 were assessed using mass spectrometry shotgun proteomics. Proteins extracted from experiment #2 were assessed using western blot analysis to validate the expression of candidate proteins, CALR, NUCB2 and SET.

To investigate how primary HBECs interact with *A*. *fumigatus* conidia, differentiated ALI cultures (Fig 1) were exposed to GFP-expressing *A*. *fumigatus* conidia. The extent of conidial internalization was assessed by visualizing differentially-stained conidia using confocal microscopy. Well-differentiated ALI cultures of primary HBECs had few conidia bound 6 hours post-exposure and less than 1% were estimated to be internalized as shown in the representative image in S1 Fig. At 24 hours post-exposure, hyphae formation by bound conidia was observed (S2 Fig); therefore, mRNA transcript and proteomic expression studies were conducted using cultures at 6 hours post-exposure to capture the early molecular response of the bronchial epithelial cells (Fig 2). This time-point is consistent with our previously published studies using submerged monolayer cultures of transformed bronchial epithelial cells (16HBE14o-) [8] and primary airway epithelial cells [51].

### NanoString analysis identified differential abundance of mRNA transcripts regulating apoptosis and complement and coagulation pathways in primary HBECs grown in ALI 6 hours post-exposure to *A*. *fumigatus* conidia

**Fig 3:**
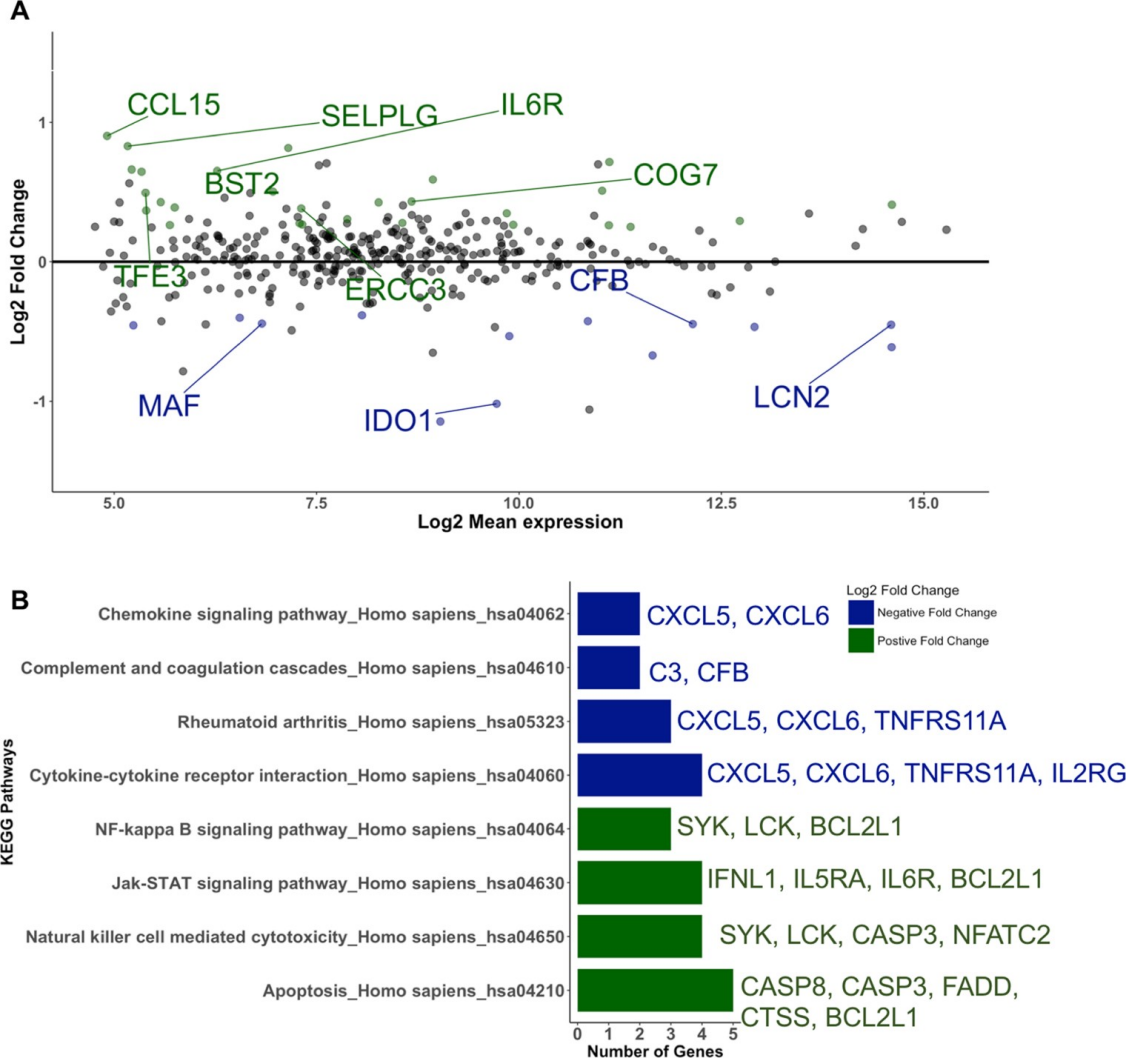
NanoString’s immune profiling panel revealed differentially expressed mrna transcripts. (A) MA plot of mRNA transcripts expressed in ALI cultures of primary HBECs 6 hours post-exposure to *A*. *fumigatus*. 41 mRNA transcripts were differentially expressed upon exposure to conidia (P-value < 0.05). Of these, 28 mRNA transcripts were up-regulated (green) and 13 mRNA transcripts were down-regulated (blue) upon exposure to conidia. 11 mRNA transcripts were significant under BH-FDR < 0.30 (labeled mRNA transcripts). (B) Enrichr identified enriched KEGG pathways for up-regulated (green) and down-regulated (blue) mRNA transcripts upon exposure to *A*. *fumigatus* conidia (Fisher exact test, Adjusted P-value < 0.05). 28 mRNA transcripts were up-regulated (positive fold change) upon exposure and green bars are corresponding to the enriched pathways. 5 mRNA transcripts (CASP8, CAP3, FADD, CTSS, and BCL2L1) were associated with Apoptosis. 13 mRNA transcripts were down-regulated (negative fold change) upon conidia exposure, associated with KEGG pathways such as cytokine-cytokine receptor interaction and complement and coagulation cascades.

We analyzed the transcriptome of immune related genes of primary HBECs grown in ALI upon interaction with *A*. *fumigatus* conidia. ALI cultures of both control and infected samples were treated identically, except for the addition of *A*. *fumigatus* conidia to the infected samples. We identified 41 mRNA transcripts that were differentially expressed (P-value < 0.05) at 6 hours post-exposure in the infected samples (S1 Table), of which 11 mRNA transcripts were significant under BH-FDR < 0.30 (Fig 3A). Compared to control ALI cultures, 28 mRNA transcripts were up-regulated and 13 mRNA transcripts were down-regulated. The up-regulated mRNA transcript with maximum Log2 fold change (Log2 fold change = 0.9027, P-value = 0.0092, BH-FDR = 0.2998) was CCL15 (C-C Motif Chemokine Ligand 15). For the down-regulated differentially expressed mRNA transcripts, CXCL5 (Chemokine (C-X-C motif) Ligand 5) had the maximum Log2 fold change (Log2 fold change = -1.1465, P-value = 0.0320, BH-FDR = 0.3315)

To determine major biological themes associated with up- and down-regulated mRNA transcripts, KEGG pathway enrichment analysis was conducted in Enrichr (http://amp.pharm.mssm.edu/Enrichr/) [53,54]. The 28 up-regulated mRNA transcripts were enriched for KEGG pathways related to apoptosis, natural killer cell mediated cytotoxicity, JAK-STAT signaling pathway and NF-kappa B signaling pathway (Fig 3B), whereas the 13 down-regulated mRNA transcripts were mainly enriched for cytokine-cytokine receptor interaction and genes involved in the complement and coagulation cascades (Fig 3B).

**Fig 4:**
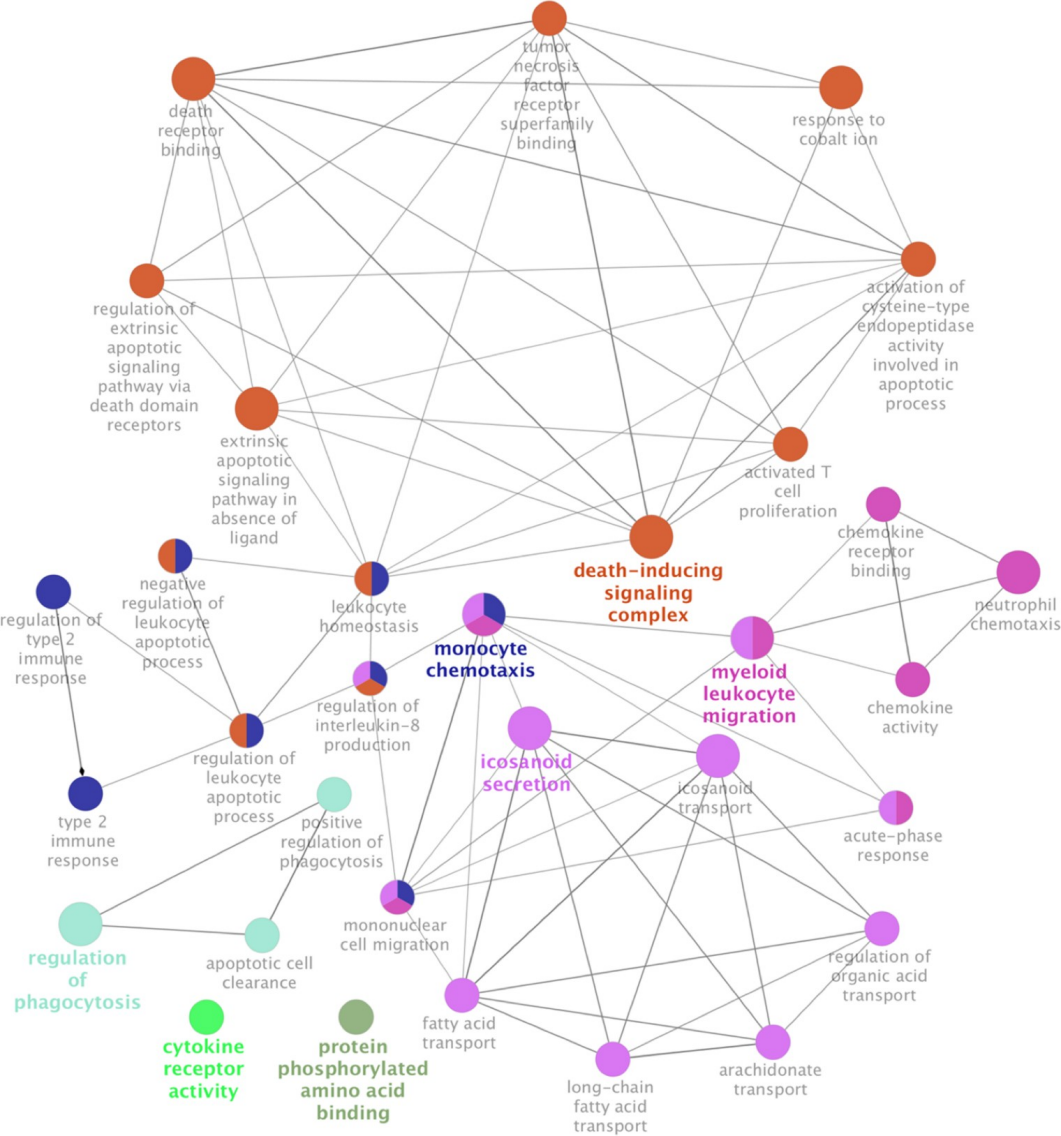
Gene ontology enrichment analysis of differentially expressed mRNA transcripts upon exposure to *A*. *fumigatus*. Functionally Grouped network of Gene Ontology enrichment analysis of differentially expressed mRNA transcripts in ClueGO App (P-value < 0.1). The differentially expressed mRNA transcripts were enriched in death-inducing signaling complex, myeloid leukocyte migration, monocyte chemotaxis, icosanoid secretion, regulation of phagocytosis, cytokine receptor activity, and protein phosphorylated amino acid binding.

Gene Ontology (GO) enrichment analysis using the Cytoscape plug-in, CLUEGO, showed that differentially expressed mRNA transcripts were enriched for death-inducing signaling cellular component, chemokine activity for molecular function, and icosanoid secretion, monocyte chemotaxis, myeloid leukocyte migration, regulation of phagocytosis were the major biological processes (Fig 4).

### Mass spectrometry shotgun proteomics analysis revealed that proteins regulating the secretory pathway were differentially expressed in ALI cells 6 hours post-exposure to *A*. *fumigatus*

**Fig 5:**
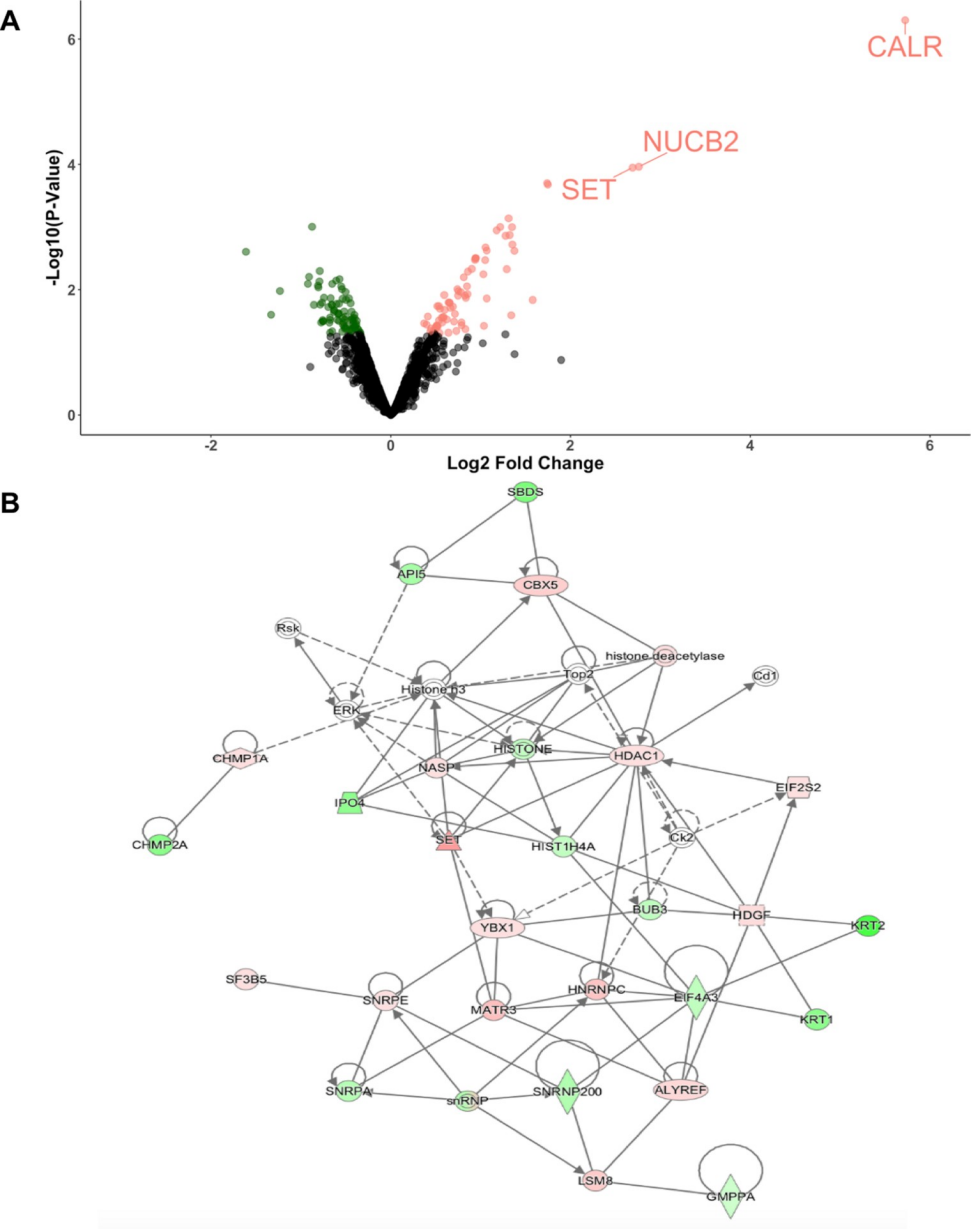
Shotgun proteomics revealed differentially expressed proteins. (A) Volcano plot of 1793 quantified proteins. Differential abundance analysis showed that 153 proteins were differentially expressed upon 6 hours post-exposure to *A*. *fumigatus* (P-value < 0.05). Of these 153, 73 were up-regulated (pink) and 80 were down-regulated (green). Three proteins, SET, NUCB2 and CALR, had a fold-change greater than 2. (B) Network analysis of 153 proteins using Ingenuity pathway analysis revealed that the top network was related to cell cycle, gene expression and tissue morphology (Score = 53).

**Fig 6:**
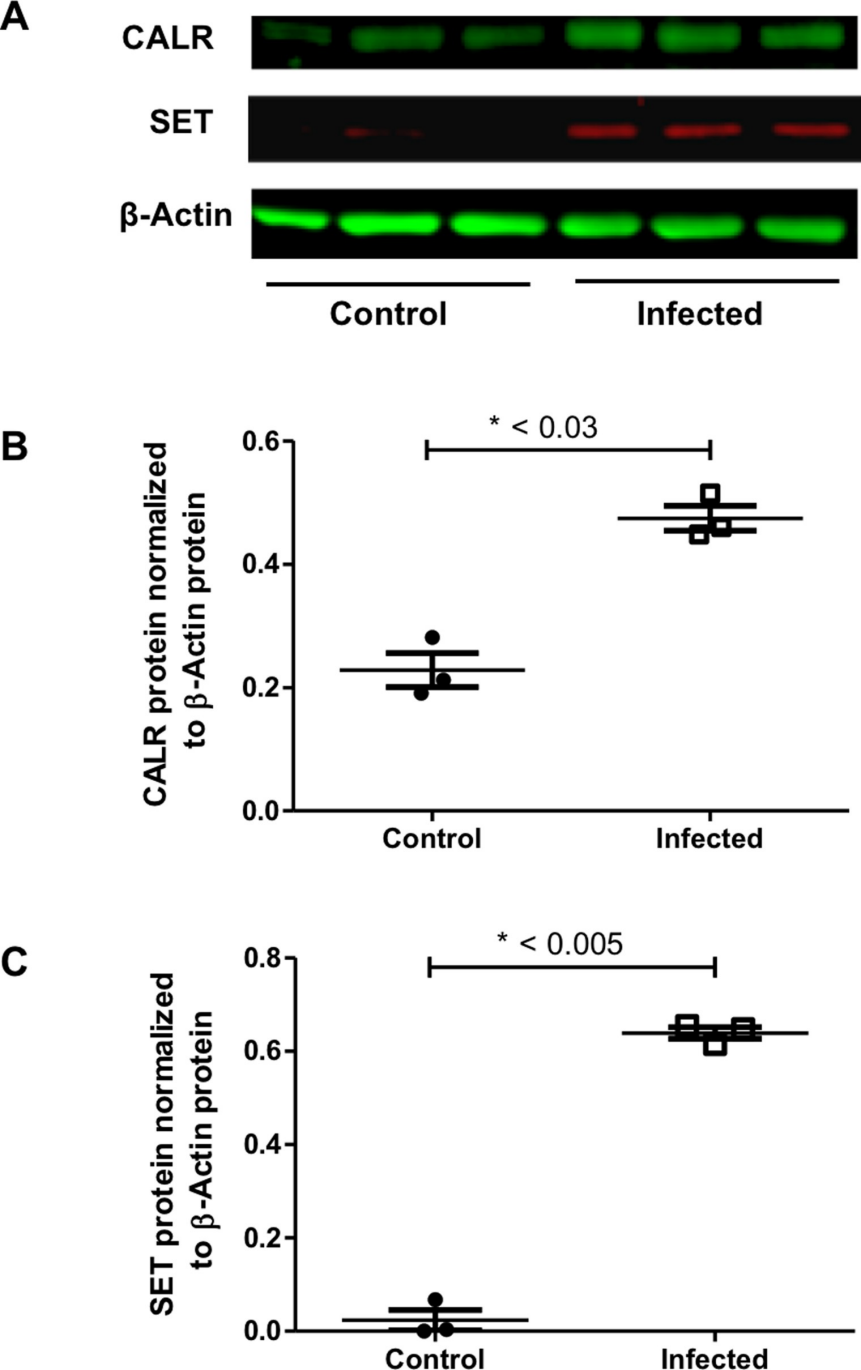
CALR and SET protein expression in ALI cultures of primary HBECs with and without *A*. *fumigatus*. (A) Band intensity for CALR, SET and β-Actin proteins after LI-COR Odyssey imaging is shown. (B) CALR (n = 3, t-test p < 0.03) was significantly up-regulated in infected (with *A*. *fumigatus* conidia suspended in PBS, n = 3) samples compared to control (PBS alone, n = 3) samples. (C) SET (n = 3, t-test p < 0.005) was significantly up-regulated in infected samples compared to control samples.

To elucidate the proteomic response of the primary HBECs to conidial exposure, proteins differential expressed upon exposure to *A*. *fumigatus* (n = 3 each for control and infected) was analyzed using Liquid Chromatography-Tandem Mass Spectrometry. In total, 2875 proteins were identified, of which 1793 proteins were quantified (at least 2 out of 3 quantification events). Differential expression analysis in LIMMA using normalized ratios of Heavy (infected) to Light (control) protein samples revealed that 153 proteins were differentially expressed in infected samples (S2 Table) and that 22 of these proteins were significant under BH-FDR < 0.30. Compared to control samples, 73 proteins were up-regulated and 80 proteins were down-regulated 6 hours post-exposure to *A*. *fumigatus* conidia (Fig 5A). Three proteins, CALR (Calreticulin), NUCB2 (Nucleobindin 2) and SET (SET nuclear proto-oncogene), had fold-changes greater than 2 (Fig 5A). Of these 3 proteins, CALR had the highest fold change of 5.723. These three proteins were selected for further analysis using western blot analysis (CALR and SET are depicted in Fig 6).

Ingenuity pathway analysis was conducted to analyze the top networks associated with differentially expressed proteins, and IPA score was used to assess the likelihood of finding the input of genes together in the network [55]. The top network was related to cell cycle, gene expression and tissue morphology (Fig 5B). Overall, the top 5 networks were associated with cell death, protein synthesis and post-translational modifications (Table 1).

**Table 1:** Top 5 networks identified using ingenuity pathway analysis (IPA) for differentially expressed proteins (P-value < 0.05).

Top 5 networks	Score
Cell Cycle, Gene Expression, Tissue Morphology	53
Cancer, Cell Death and Survival, Organismal Injury and Abnormalities	44
Cellular Development, Protein Synthesis, Gene Expression	34
Post-Translational Modification, Protein Degradation, Endocrine System Disorders	27
Drug Metabolism, Protein Synthesis, Renal Damage	23

Pathway enrichment analysis for differentially expressed proteins was conducted in Enrichr using the Reactome database (http://amp.pharm.mssm.edu/Enrichr/) [34,54]. Translation, Metabolism of proteins, 3`-UTR mediated translational regulation, Nonsense mediated decay, Major pathway of rRNA processing in the nucleolus and Metabolism of amino acids and derivatives were enriched pathways for the differentially expressed proteins (Table 2).

**Table 2:** Enriched Reactome pathways for differentially expressed proteins (P-value < 0.05) identified using Enrichr.

ID	Term	Overlap	P-value	Adjusted P-value
R-HSA-72766	Translation	22/151	3.90703E-22	2.53566E-19
R-HSA-392499	Metabolism of proteins	43/1074	7.60833E-20	2.4365E-17
R-HSA-157279	3' -UTR-mediated translational regulation	18/106	1.57142E-19	2.4365E-17
R-HSA-168255	Influenza Life Cycle	17/136	3.60394E-16	1.7992E-14
R-HSA-1799339	SRP-dependent co-translational protein targeting to membrane	16/107	1.47713E-16	8.71507E-15
R-HSA-927802	Nonsense-Mediated Decay (NMD)	16/106	1.26443E-16	8.20614E-15
R-HSA-6791226	Major pathway of rRNA processing in the nucleolus	14/166	3.73975E-11	8.66821E-10
R-HSA-71291	Metabolism of amino acids and derivatives	17/335	8.42701E-10	1.82304E-08
R-HSA-72163	mRNA Splicing—Major Pathway	10/134	8.2771E-08	1.6787E-06
R-HSA-597592	Post-translational protein modification	14/521	4.86567E-05	0.0007702
R-HSA-199977	ER to Golgi Anterograde Transport	7/131	6.75186E-05	0.001043323
R-HSA-381038	XBP1(S) activates chaperone genes	4/53	0.000720512	0.009543107

To determine functional processes associated with 73 up-regulated proteins and 80 down-regulated proteins, gene ontology (GO) enrichment analysis was conducted using Enrichment analysis tool in the Gene Ontology Consortium (http://geneontology.org) [56,57]. Some of the enriched terms for up-regulated proteins upon exposure to *A*. *fumigatus* were cadherin binding, spliceosomal complex, endoplasmic reticulum-Golgi intermediate compartment and translation initiation factor activity (BH-FDR < 0.05) (Table 3). The down-regulated proteins were associated with extracellular exosome, non-sense mediated decay, rRNA processing, structural constituent of ribosome, extracellular matrix, oxidation-reduction process (BH-FDR < 0.05) (Table 4). GO terms related to translation and splicing were enriched in both up- and down-regulated proteins.

**Table 3:** Enriched Gene Ontology (GO) terms for 73 up-regulated differentially expressed proteins (P-value < 0.05) identified using Gene Ontology Consortium (MF = Molecular Function; CC = Cellular Component; BP = Biological Processes).

GO	Term	Overlap	P-value	FDR
MF	cadherin binding (GO:0045296)	11/295	9.19E-09	2.12E-05
CC	cell-cell adherens junction (GO:0005913)	5/92	2.31E-05	2.11E-03
CC	ruffle (GO:0001726)	6/165	3.03E-05	2.24E-03
MF	protein complex binding (GO:0032403)	13/796	4.19E-06	2.42E-03
CC	spliceosomal complex (GO:0005681)	6/188	6.14E-05	3.46E-03
CC	endoplasmic reticulum-Golgi intermediate compartment (GO:0005793)	5/115	6.42E-05	3.51E-03
CC	U12-type spliceosomal complex (GO:0005689)	3/27	1.57E-04	7.70E-03
CC	precatalytic spliceosome (GO:0071011)	3/29	1.90E-04	8.90E-03
CC	peptidase complex (GO:1905368)	4/93	3.76E-04	1.53E-02
MF	translation initiation factor activity (GO:0003743)	4/52	4.45E-05	1.86E-02
MF	cytoskeletal protein binding (GO:0008092)	12/884	6.14E-05	2.18E-02
CC	centriolar subdistal appendage (GO:0120103)	2/9	6.53E-04	2.45E-02
CC	centriole (GO:0005814)	4/127	1.16E-03	3.78E-02
MF	protein homodimerization activity (GO:0042803)	11/811	1.29E-04	4.27E-02
CC	spliceosomal snRNP complex (GO:0097525)	3/63	1.61E-03	4.55E-02
CC	cell cortex part (GO:0044448)	4/139	1.61E-03	4.60E-02

**Table 4:** Enriched Gene Ontology (GO) terms for 80 down-regulated differentially expressed proteins (P-value < 0.05) identified using Gene Ontology Consortium (MF = Molecular Function; CC = Cellular Component; BP = Biological Processes).

GO	Term	Overlap	P-value	FDR
CC	extracellular exosome (GO:0070062)	43/2757	4.67E-17	8.94E-14
BP	nuclear-transcribed mRNA catabolic process, nonsense-mediated decay (GO:0000184)	14/119	1.40E-16	2.17E-12
BP	SRP-dependent cotranslational protein targeting to membrane (GO:0006614)	12/92	8.61E-15	4.46E-11
BP	translational initiation (GO:0006413)	13/143	3.96E-14	7.70E-11
BP	rRNA processing (GO:0006364)	14/261	3.42E-12	3.12E-09
MP	structural constituent of ribosome (GO:0003735)	12/169	6.19E-12	2.85E-08
CC	cytosolic large ribosomal subunit (GO:0022625)	7/65	1.34E-08	1.22E-06
CC	cytosolic small ribosomal subunit (GO:0022627)	5/47	1.86E-06	1.15E-04
MP	protein binding (GO:0005515)	67/11523	1.16E-06	1.34E-03
MP	rRNA binding (GO:0019843)	5/59	5.27E-06	4.05E-03
CC	spliceosomal complex (GO:0005681)	6/188	1.17E-04	6.03E-03
CC	focal adhesion (GO:0005925)	8/394	1.82E-04	8.95E-03
CC	extracellular matrix (GO:0031012)	9/549	3.40E-04	1.42E-02
BP	oxidation-reduction process (GO:0055114)	13/949	9.16E-05	1.85E-02
CC	integral component of membrane (GO:0016021)	9/68	4.55E-04	1.86E-02
BP	carbohydrate metabolic process (GO:0005975)	9/472	1.13E-04	2.25E-02
CC	nuclear lumen (GO:0031981)	28/3938	1.06E-03	3.97E-02

### Western blot analysis showed significant changes in the expression of CALR and SET in ALI cultures of primary HBECs 6 hours post-exposure to *A*. *fumigatus*

To validate the expression of the top three differentially expressed proteins, CALR, NUCB2 and SET, with fold changes greater than 2, western blot analysis was conducted. CALR and SET, but not NUCB2 (results not shown), proteins were significantly up-regulated in infected samples compared to control samples (P-value < 0.05) (Fig 6).

## Discussion

In this study, NanoString nCounter platform and shotgun proteomics were used for the first time to analyze the transcriptomics and proteomics of primary HBECs grown in ALI upon exposure to *A*. *fumigatus* conidia for 6 hours. We demonstrated that primary HBECs grown in ALI are capable of phagocytosing conidia; however, the proportion of bound conidia internalized by these differentiated airway cells was less than 1%, even after 6 hours post-exposure (S1 Fig). After 24 hours, bound conidia had germinated and formed hyphae (S2 Fig). Two previous studies have quantified uptake of *A*. *fumigatus* conidia by 14-day old ALI cultures: Botterel et al. (2008) showed that human nasal epithelial cells internalized 21.8 ± 4.5% of bound conidia after 4 hours, and a study by Khoufache et al., (2010) found that 21.9 ± 1.4% of conidia were internalized by porcine tracheal epithelial cells after 8 hours [40,41]. We believe that the lower rate of conidial uptake in our study may have been due to the more differentiated state of the ALI cultures; however further characterization of the interaction between *A*. *fumigatus* conidia and primary HBECs grown in ALI is required. To our knowledge, no data has been published on phagocytosis of live conidia by fully-differentiated ALI cultures of human bronchial epithelial cells (21–28 days old). Beisswenger et al. reported that primary HBECs grown in ALI are activated by resting conidia resulting in activation of IFN-β signaling pathway but no phagocytosis results were reported using live *A*. *fumigatus* conidia [58]. The results we obtained in differentiated ALI cultures *in vitro* are supported by those of Rammaert et al., who measured internalization of fungal conidia by the bronchial epithelium of mice *in vivo* using transmission electron microscopy. They found no evidence of phagocytosis of either *A*. *fumigatus* or *Lichtheimia corymbifera* conidia in bronchial epithelial cells in the first 18 hours post-exposure [43]. Hence, the effect of fungal conidia on early molecular response is mediated either by binding of *A*. *fumigatus* conidia to host bronchial epithelia, or alternatively, by interaction of molecules from the surface of the conidia that initiate the host immune response.

Previously, our laboratory has reported transcriptional responses to *A*. *fumigatus* conidia by 16HBE14o-, an SV-40-transformed human bronchial epithelial cell line, and primary human airway epithelial cells that were grown in submerged monolayers. After 6 hours post-exposure to *A*. *fumigatus*, we found enrichment of ontologies related to the innate immune response [8,51], but there were discrepancies between the transcriptional responses of the cell line and primary cells. Hence, for this current study, we employed a differentiated *in vitro* model of primary cells that more closely mimics the *in vivo* epithelium to study the host immune response using transcriptomics. In addition, we utilized untargeted shotgun proteomics to study the host molecular response upon exposure to *A*. *fumigatus* conidia for 6 hours.

The precise and sensitive NanoString nCounter platform [59,60] was used to profile 730 immune genes in primary HBECs upon exposure to *A*. *fumigatus* conidia. Enrichment of pathways and gene ontologies associated with innate immune response more closely matched our previous results from submerged primary airway epithelial cells rather than the transformed cell line. Interestingly, mRNA transcripts related to complement and coagulation cascades such as Complement C3 (C3) and Complement Factor B (CFB) were down-regulated upon *A*. *fumigatus* infection. This is also consistent with previous findings by Behnsen et al., suggesting that *A*. *fumigatus* may bind to complement regulators as a way of evading host attack mediated by the complement system [61]. Neutrophil chemoattractants, such as C-X-C Motif Chemokine Ligand 5 (CXCL5) and C-X-C Motif Chemokine Ligand 6 (CXCL6), were also down-regulated upon exposure to *A*. *fumigatus* [62]. Additionally, Annexin-1 (ANXA1), known to limit neutrophil recruitment and production of pro-inflammatory mediators [63], was up-regulated upon exposure to *A*. *fumigatus* as well. These genes regulating neutrophil recruitment are likely to play an important role in IA, since neutrophils have been shown to control germination of *A*. *fumigatus in vivo* [64]. Previously, pro-inflammatory cytokines and chemokines have been reported to be up-regulated in transcriptomics studies of airway epithelial cells upon exposure to *A*. *fumigatus* [8,34,51]. Hence, further studies need to be conducted to elucidate their role in host-pathogen interaction.

Additionally, mRNA transcripts regulating T cell proliferation and the T_H_1/ T_H_2 response were also differentially expressed in infected cells. These included MAF BZIP Transcription Factor (MAF), Interferon-Lambda 1 (IFN-λ or IL-29), and Indoleamine 2, 3-dioxygenase (IDO-1). MAF was also the most significant differentially expressed mRNA transcript in the Immune Profiling Panel analysis (P-value = 0.00172; BH-FDR = 0.29984). It is a T_H_2 associated proto-oncogene, involved in the production of Interleukin-4 (IL-4), a T_H_2 associated cytokine that promotes differentiation of naïve CD4+ T cells into IL-4 producing T_H_2 cells [65]. ABPA occurs in patients with skewed pulmonary immune responses, such as those found in asthma or cystic fibrosis, in whom the fungus elicits a strong T_H_2 response [66]. Hence, downregulation of MAF upon exposure to *A*. *fumigatus* in primary HBECs from healthy individuals, may indicate a protective response to the fungus. IFN-λ was up-regulated upon exposure to *A*. *fumigatus*. Along with having anti-viral properties, IFN-λ is an inhibitor of T_H_2 responses as well, limiting the secretion of IL-4, IL-5 and IL-13 cytokines [67]. IDO-1, down-regulated upon exposure to *A*. *fumigatus*, is known to catalyze the first step in the degradation of tryptophan [68]. High expression of IDO-1 is thought to be associated with downregulation of immune response as degradation of tryptophan results in inhibition of T-cell proliferation and apoptosis [69].

Furthermore, mRNA transcripts associated with apoptosis, such as CASP3 (Caspase 3), CASP8 (Caspase 8), FADD (Fas-associated protein with death domain), LCN2 (Lipocalin 2), and BCL2L1 (BCL2 Like 1), were also differentially expressed, indicating that the interaction of conidia with epithelial cells may promote apoptosis. This may be due to a secondary metabolite produced by *A*. *fumigatus* called gliotoxin. Gliotoxin has been shown to induce pathways associated with apoptosis in human bronchial epithelial cells by the Bcl-2 pathway as well as *via* a caspase-dependent mechanism [70,71]. However, genes such as CASP8, BCL2L1 involved in such pathways have been shown to have a non-apoptotic role as well [71]. Specifically, cell stress could also induce autophagy, a pro-survival mechanism, which can allow cells to survive prolonged stress caused by infectious agents or nutrient deprivation [72].

We also quantified the changes to the proteome of primary HBECs as a consequence of exposure to *A*. *fumigatus* conidia. Proteins regulating the secretory pathway were differentially expressed 6 hours post-exposure and the top three proteins (Fold change > 2), CALR, NUCB2 and SET, are involved in protein folding and quality control as well as calcium metabolism. To help validate the physiological relevance of results from shotgun proteomics, we used western blot analysis to evaluate top three differentially expressed proteins, CALR, NUCB2 and SET. Western blot analysis confirmed significant differential expression of CALR and SET (Fig 6), but not NUCB2 (results not shown).

CALR, an intracellular chaperone, has been shown to mediate phagocytosis of *A*. *fumigatus* conidia by forming a calreticulin-CD91 complex as well [73]. Additionally, up-regulation of CALR has also been associated with increased Ca^2+^ storage capacity in the endoplasmic reticulum (ER) as well as increased sensitivity to apoptosis [74,75]. NUCB2 is a calcium binding protein [76] whereas SET is a multi-tasking protein involved in processes such as apoptosis, transcription, nucleosome assembly and histone chaperoning [77]. Differentially expression of proteins related to calcium metabolism and binding could be due to ER stress, which can promote cell-death or cell-survival as well as release of Ca^2+^ from the ER [78]. Unfolded Protein Response (UPR), an ER stress response, can also be activated and can result in up-regulation of molecular chaperones to increase protein folding, halting protein translation, degradation of misfolded proteins, and regulation of cell death and survival as well [79]. Since UPR can result in expression of chaperones, other than CALC, Heat shock protein 90α (HSP90AA1 or HSP90α) was also up-regulated upon exposure to *A*. *fumigatus*. It is an isoform of molecular chaperone Hsp90, and has been previously shown to be up-regulated in the presence of stress [80]. It was one of the 8 differentially expressed genes that were also differently expressed in our previous study by Gomez et al. [8]. Even though it was down-regulated in 16HBE14o- upon exposure to *A*. *fumigatus* conidia, replication of this gene indicates that it may play an important role in the host-pathogen interaction.

High levels of Ca^2+^ have also been shown to attenuate nonsense mediated decay (NMD) [81]. This was evident in the gene ontologies enriched for down-regulated proteins as the majority of proteins associated with NMD were down-regulated upon exposure to conidia. Additionally, proteins regulating other translational processes were both up-regulated and down-regulated in primary HBECs upon exposure to A. *fumigatus*, as indicated by enriched pathways and gene ontologies. For example, proteins associated with eukaryotic translation initiation (EIF) such as EIF factor-3 Subunit J (EIF3J), EIF Factor 2 Subunit Beta (EIF2S2) were up-regulated upon exposure to *A*. *fumigatus* conidia. On the contrary, the majority of ribosomal proteins including those from the 60S large subunit (RPL) and 40S small subunit (RPS), such RPL3, RPS8, RPS5, were down-regulated upon exposure to A. *fumigatus*, which could serve to lower the overall protein traffic into the ER. Ribosomal proteins can play a role in regulating apoptosis, cell cycle and cell proliferation [82]. Proteins regulating cellular processes such as cell cycle progression were also up-regulated upon exposure to *A*. *fumigatus*, including SET and HSP90AA1, as previously mentioned. However, other proteins that were up-regulated upon exposure to *A*. *fumigatus* and that are essential for cell cycle progression and formation of cilia included Cenexin (ODF2), Dynactin (DCTN1), FGFR1 Oncogene Partner (FGFR1OP or FOP), Histone deacetylase 1 (HDAC1) and Tumor Protein P53 Binding Protein 1 (TP53B1). Hence, genes regulating cell cycle and formation of cilia may be essential to prevent fungal invasion of host tissue as well.

Another key process that was enriched in the down-regulated proteins was cellular iron homeostasis (Ferritin Light Chain (FTL), Superoxide Dismutase (SOD1), ATPase H+ Transporting V0 Subunit D1 (ATP6V0D1)). We have previously shown that genes associated with iron uptake were up-regulated in *A*. *fumigatus* [51] and a number of studies have shown that iron acquisition is important for *A*. *fumigatus* virulence [83,84]. Specifically, FTL was also up-regulated in our previous study of 16HBE14o- cells upon exposure to *A*. *fumigatus* conidia [8]. However, down-regulation of proteins such as FTL, an iron-binding protein, increases the amount of free iron available to the fungus [85]. Since free iron is a catalytic agent for the Fenton reaction that generates free radicals, it can result in oxidative damage in epithelial cells [86].

In summary, we employed an ALI model that closely mimics the bronchial epithelial barrier in the conductive zone of the respiratory tract to study proteomics and transcriptomics of bronchial epithelial cells upon exposure to *A*. *fumigatus* conidia. The major pathways that were up-regulated by conidial exposure included apoptosis/autophagy, translation, unfolded protein response, and cell cycle. In contrast, complement and coagulation pathways, iron homeostasis, non-sense mediated decay, and rRNA binding pathways were down-regulated upon exposure to conidia. We have validated the up-regulation of CALR and SET in ALI cultures of primary HBECs 6 hours post-exposure to *A*. *fumigatus*. One limitation of this study was the small sample size, and we were also unable to identify airway epithelial cell responses independently of other cells types such as basal, mucus-producing goblet or ciliated cells in these ALI cultures. Further characterization of the host-pathogen interaction in ALI cultures of primary HBECs post-exposure to *A*. *fumigatus* conidia will be conducted in future studies. Our ultimate goal is to use ALI cultures of primary HBECs to better understand how host pathology, in particular asthma or cystic fibrosis, influences their transcriptomic and proteomic responses to *A*. *fumigatus* conidia.

## Conclusion

We have demonstrated that, unlike submerged monolayers, ALI cultures of primary HBECs were estimated to internalize less than 1% of bound *A*. *fumigatus* conidia. Both transcriptional and proteomics analyses of HBECs revealed that genes related to cell cycle regulation, apoptosis/autophagy, iron homeostasis, calcium metabolism, complement and coagulation cascades, translation, ER stress and UPR are enriched in the early immune response upon interaction with *A*. *fumigatus* conidia. These data reveal that ALI cultures of primary bronchial human epithelial cells can provide novel insights into the mechanisms of different types of diseases associated with this opportunistic fungal pathogen.

## Methods

### ALI cultures of primary HBECs

Human lungs of de-identified healthy donors, deemed unsuitable for transplantation and donated to medical research, were obtained from the International Institute for the Advancement of Medicine (Edison, NJ) for primary cell isolation as per approval by the Research Ethic Board (REB) of University of British Columbia / Providence Healthcare (REB# H00-50110). Bronchial epithelial cells were isolated by protease digestion as described by Gray and colleagues and cultured in bronchial epithelial growth medium (Lonza, Mississauga, ON, CC-3170) at 37°C in 5% CO_2_. ALI cultures of primary HBECs were generated using cells at passage one or two in ALI PneumaCult medium (Stemcell Technologies, Vancouver, BC, Item# 05001). The cultures were grown for 21–28 days on 12-well (Griener Bio-One, Item# 665180) or 24-well (Greiner Bio-One, Item# 662160) plates to generate a pseudostratified epithelium [87,88]. The ALI cultures contained basal, mucus-producing goblet and ciliated cells. Barrier function of epithelial cells was assessed by measuring Trans-Epithelial Electrical Resistance (TEER) values (S3 Table). Previously, TEER values have been used to assess the integrity of tight junctions and cellular barriers for ALI cultures [89,90]; hence due to large differences in TEER values among ALI cultures grown in 12-well format (S3 Table), TEER values of all 12 samples were included as a covariate during differential expression analysis for transcriptional analysis. For the 6 cultures grown in 12-well format assessed using proteomics, the two low TEER samples were paired in duplexes during dimethyl labeling.

### Immunohistochemical (IHC) analysis

Primary HBECs grown as differentiated ALI cultures were fixed in 10% formalin, embedded in paraffin, and sectioned into 4 μm slices. Sections were deparaffinized in CitriSolv (Fisher Scientific, Toronto, ON, 22-143-975) as per the standard protocol. The differentiation status of ALI cultures was confirmed by Haemotoxylin and Eosin (H&E), a standard immuno-staining technique, where hematoxylin was used for counter-staining of the nuclei. Matched ALI section was processed for periodic acid Schiff (PAS) reagent for quantifying mucus secretion based on the staining of carbohydrates. For CK-5 immunostaining, 4 μm thick ALI section was de-waxed, rehydrated, and subjected to heat-induced antigen retrieval in citrate buffer (pH 6.0, Invitrogen). Tissue sections were blocked in blocking buffer consisting of Tris-buffered saline (TBS) + 1% BSA for 30 minutes at room temperature. These sections were then incubated with mouse anti-human CK-5 antibody (1:2000 dilution) or normal mouse immunoglobulin G (IgG) as a negative control (Abcam, Item# AB181491, and BioRad, Item# MCA928, respectively) in blocking buffer overnight at 4°C. Tissues were washed in TBS and treated with Biotin-free MACH 4 AP-Polymer Detection kit (Bio-care Medical, Item# M4U536) containing alkaline phosphatase, followed by Warp Red Chromogen (Biocare Medical, Item# WR806) substrate for 7 minutes at room temperature. Cell nuclei were counterstained with hematoxylin. Brightfield images (Nikon Eclipse E600 microscope, Nikon, El Segundo, CA, USA) of ALI sections for above mentioned staining were digitally captured (SpotFlex camera; Diagnostic Instruments Sterling Heights, MI, USA).

### Culture conditions and preparation of *A*. *fumigatus* conidia

All experiments were performed using a green fluorescent protein (GFP)-expressing strain of *A*. *fumigatus* derived from ATCC 13073 (American Type Culture Collection, Manassas, VA), developed by Waslynka and Moore [35]. Briefly, the strain was transformed by electroporation with a plasmid containing the codon-optimized *sgfp* gene and the construct yielded stable, high expression of GFP in both conidia and hyphae. The GFP-transformed *A*. *fumigatus* strain was grown on yeast-agar-glucose media at 30°C until sporulation. Mature conidia were harvested by gently scrubbing the plates using sterile cotton swabs with phosphate-buffered saline plus 0.05% Tween-20 (PBS-T). The conidial suspension was filtered through sterile glass wool, vortexed, pelleted and re-suspended in 1 ml PBS. The suspension was washed twice with PBS to remove any trace of PBS-T prior to quantification using a hemocytometer.

### RNA and protein preparation

ALI cultures of primary HBECs were incubated with PBS alone (control, n = 3) or *A*. *fumigatus* conidia suspended in PBS, MOI = 10 (infected, n = 3). Cells were exposed to conidia on the apical side for 6 hours at 37°C in two separate experiments. The basal sides of ALI cultures were incubated with ALI PneumaCult medium. For each experiment, 6 hours post-exposure to PBS or PBS+conidia, culture supernatants from both the apical and basal sides were removed. Both sides of the membrane were washed twice with sterile PBS to remove any unbound conidia. The membrane of each insert was detached with a pipette tip and collected in a microcentrifuge tube. Lysis Buffer Q (300 μl) was added to each tube according to the standard operating protocol of the RNA/DNA/Protein Purification Plus Micro Kit (Norgen Biotek Corp, Item# 51600) and membranes were incubated at -80°C. DNA, RNA and protein were extracted as directed by the standard protocol noted above.

### NanoString nCounter gene expression analysis

RNA yield from each sample was determined using NanoDrop ND-100 spectrophotometer (ThermoFisher Scientific, Wilmington, DE) and RNA integrity was determined using a 2100 Bioanalyzer (Agilent Technologies, Santa Clara, CA). mRNA transcript abundance was analyzed from 100 ng of extracted RNA using the NanoString nCounter Immune Profiling panel (NanoString Technologies, Seattle, WA) from two separate experiments (n = 3 for both control and infected in each experiment). The Immune Profiling panel consisted of 770 genes (730 well-annotated immune genes and 40 housekeeping genes). Briefly, 70 μl of hybridization buffer was added to Reporter CodeSet to prepare the master mix. To set up the hybridization reactions, each sample tube contained 8 μl of master mix and 5 μl of extracted RNA sample. Capture ProbeSet (2 μl) was added to each tube and samples were hybridized at 65°C for 19 hours. The hybridized samples were analyzed using FLEX system’s nCounter Prep Station using the high sensitivity protocol, and the cartridge was scanned using Maximum resolution (Max FOV) in the nCounter Digital Analyzer to generate RCC files. Raw data (S4 Table) was normalized using the positive control genes in the statistical program R, and lowly expressed genes were filtered. Total sum normalization was performed before combining the data from both experiments. Surrogate Variable Analysis (sva) package [version 3.26.0] was used to perform batch correction in R, prior to differential abundance analysis.

### Protein solubilization and duplex dimethylation labeling

Extracted proteins from control (PBS alone, n = 3) and infected ALI samples (with *A*. *fumigatus* conidia suspended in PBS, n = 3) grown in 12-well format (S3 Table) were solubilized in a small volume of 6 M urea/2 M thiourea (in 10 mM Hepes, pH 8.0) and proteins were then precipitated using the ethanol-acetate method [91]. The protein concentrations of the samples were measured by the Bradford assay, followed by digestion in solution using Trypsin and Lys-C according to reference [91]. Digested peptides were purified and concentrated on C18 STAGE-tips, eluted in 80% acetonitrile, 0.5% acetic acid, and dried in a vacuum concentrator (Eppendorf) [92]. Dried peptides were re-suspended in 100 mM Triethylammonium bicarbonate and chemical di-methylation labeling was performed using light (CH2O) or heavy (13CD2O) isotopologues of formaldehyde. The light label was used for control samples and the heavy label for the infected samples. Light sodium cyanoborohydride solution (1M) was added to the light labeled samples and 1 M heavy sodium cyanoborodeuteride to the heavy labeled samples. The samples were vortexed and incubated at ambient temperature in the dark for 90 minutes. NH_4_CL (3 M) was added to the samples after which they were incubated at ambient temperature in the dark for 10 minutes. Samples were acidifed to pH < 2.5 by adding 1% Trifluoroacetic acid (TFA). After full sodium cyanoborohydride degradation, each heavy labeled sample was combined with the light labeled sample (n = 3) and STAGE-tip purified. Eluted samples were dried and re-suspended in 2% acetonitrile in 5mM ammonium formate for subsequent fractionation. Peptides were separated offline using basic reverse phase fractionation as described previously [93].

### Liquid chromatography-tandem mass spectrometry (LC-MS/MS) and protein identification

Peptides fractions were analyzed by a quadrupole–time of flight mass spectrometer (Impact II; Bruker Daltonics) coupled to an Easy nano LC 1000 HPLC (ThermoFisher Scientific) using an analytical column that was 40–50 cm long, with a 75 μm inner diameter fused silica with an integrated spray tip pulled with P-2000 laser puller (Sutter Instruments) and packed with 1.9 μm diameter Reprosil-Pur C-18-AQ beads (Maisch, www.Dr-Maisch.com). The columns were operated at 50°C using an in-house built column heater. Buffer A consisted of 0.1% aqueous formic acid, and buffer B consisted of 0.1% formic acid and 80% (vol/vol) acetonitrile in water. A standard 90-min peptide separation was done, and the column was washed with 100% buffer B before re-equilibration with buffer A. The Impact II was set to acquire in a data-dependent auto-MS/MS mode with inactive focus fragmenting the 20 most expressed ions (one at the time at a 18-Hz rate) after each full-range scan from m/z 200 to m/z 2,000 at 5 Hz rate. The isolation window for MS/MS was 2–3 depending on the parent ion mass to charge ratio, and the collision energy ranged from 23 to 65 eV depending on ion mass and charge. Parent ions were then excluded from MS/MS for the next 0.4 min and reconsidered if their intensity increased more than five times. Singly charged ions were excluded from fragmentation. Raw mass spectrometry data was analyzed using MaxQuant 1.5.1.0. The search was performed against a database comprised of the protein sequences from Uniprot’s human entries plus common contaminants with cysteine carbamidomethylation and methionine oxidation, protein N-acetylation as fixed and variable modifications, respectively. Light and heavy dimethylation at lysine side chains and peptide N-termini were used for quantitation. Peptides and proteins identified with FDR ≤ 1% were retained for further analyses.

### Statistical analysis

Differential abundance of mRNA transcripts and proteins 6 hours post-exposure to *A*. *fumigatus* conidia was determined using least squares regression in the Linear Model for MicroArrays (LIMMA) [Version 3.30.13] package in R statistical computing program. A P-value < 0.05 was considered statistically significant for both transcriptomics and proteomics analyses. A Benjamini-Hochberg False Discovery Rate (BH-FDR) of 30% was used as well. All software packages used to perform differential expression analyses were accessed through The Comprehensive R Archive Network (CRAN) (https://cran.rproject.org/).

### Bioinformatics analysis

Pathway enrichment analyses of differentially expressed mRNA transcripts and proteins were conducted in Enrichr (http://amp.pharm.mssm.edu/Enrichr/) [53,54]. Data were analyzed through the use of Ingenuity Pathway Analysis (IPA) (QIAGEN Inc., https://www.qiagenbioinformatics.com/products/ingenuity-pathway-analysis) to assess the top networks associated with differentially expressed proteins as well [55].

The Cytoscape plug-in, ClueGo [Version 2.5.0] + CluePedia [Version 1.5.0] [94,95] was used to generate functionally grouped networks of enriched gene ontology (GO) terms associated with biological processes, molecular function and cellular components for differentially expressed mRNA transcripts (P-value < 0.01).

Gene Ontology Consortium’s PANTHER Overrepresentation Test (http://www.geneontology.org/page/go-enrichment-analysis) was also used to generate enriched GO terms for biological processes, molecular functions and cellular components of differentially expressed proteins [56,57]. Fisher’s Exact with FDR multiple test correction was used (BH-FDR < 0.05).

### Western blot analysis

Total cell lysate proteins extracted from control (PBS alone, n = 3) and infected ALI samples (with *A*. *fumigatus* conidia suspended in PBS, n = 3), grown in 24-well format (S3 Table), were used for western blot analysis according to standard techniques [96]. Twenty micrograms of reduced protein samples were electrophoresed in a 4–20% gradient SDS-PAGE gel and then transferred to a nitrocellulose membrane. The membrane was blocked for 1 hour at room temperature in Odyssey Blocking Buffer (LI-COR Biosciences, Item# 927–50000). Western immunoblots were developed in sequence using primary antibodies at 1:1000 dilution (rabbit anti SET antibody (ThermoFisher Scientific, Item# PA5-78163), mouse monoclonal anti-Calreticulin antibody (ThermoFisher Scientific, Item# MA5-15382), rabbit anti-NUCB2 polyclonal antibody (ThermoFisher Scientific, Item# PA5-78096)) and secondary antibodies at 1:10000 dilutions (anti-mouse IgG (H+L) (DyLight 800 4X PEG Conjugate, Cell Signaling Technology, Item# 5257S), anti-rabbit IgG (H+L) (DyLight 680 Conjugate, Cell Signaling Technology, Item# 5366S)). The membranes were washed 4 times with Tris-buffered Saline and Tween-20 (TBST) for 5 mins between primary and secondary antibodies, and after the secondary antibody. The membrane was then scanned on a LI-COR Odyssey 2.1 Infrared Imaging System (Lincoln, Nebraska, USA). The integrated intensity of each band was obtained using the software Image J, with subtraction of background intensity. Densitometry values for SET, CALR and NUCB2 were normalized to β-actin for equal loading. Statistical significance was obtained using paired *t* test (P-value < 0.05).

## Supporting information

S1 Fig Differential staining of extracellular and internalized conidia by anti-*A*. *fumigatus* antibody using confocal microscopy at 6 hours post-infection. GFP-expressing *A*. *fumigatus* conidia and primary HBECs grown in ALI were co-incubated for 6 hours, fixed and stained with DAPI to label cell nuclei, and a monoclonal anti-*A*. *fumigatus* antibody was used to label extracellular conidia, before visualization using confocal microscopy. One representative field is shown in the following channels: (A) wavelength 594nm for anti-*A*. *fumigatus* antibody (red); (B) wavelength 495nm for GFP (green); (C) wavelength 405nm for DAPI (blue); (D) merged GFP, anti-*A*. *fumigatus* antibody and DAPI image. Conidia not labeled by the anti-*A*. *fumigatus* antibody and only visible in the green but not the red channel were considered to be internalized by ALI cultures of primary HBECs. Field of view is 1912x1912 pixels, and scale bar is 10 μm.
(TIF)

S2 Fig Differential staining of extracellular and internalized conidia by anti-*A*. *fumigatus* antibody using confocal microscopy after 24 hours of co-incubation. GFP-expressing *A*. *fumigatus* conidia and primary HBECs grown in ALI were co-incubated for 24 hours and processed as described in Fig 2 legend. (A) wavelength 594nm for anti-*A*. *fumigatus* antibody (red); (B) wavelength 495nm for GFP (green); (C) wavelength 405nm for DAPI (blue); (D) merged GFP, anti-*A*. *fumigatus* antibody and DAPI image. Hyphae (white arrows) germinated from the bound conidia is shown. Field of view is 512x512 pixels with a zoom of 2x, and the scale bar is 10 μm.
(TIF)

S1 Table Differentially expressed mRNA transcripts in ALI cultures 6 hours post-exposure to *A*. *fumigatus* conidia (P-value < 0.05). mRNA transcripts significant under BH-FDR < 0.30 are in bold.
(DOCX)

S2 Table Differentially expressed proteins in ALI cultures 6 hours post-exposure to *A*. *fumigatus* conidia (P-value < 0.05). 8 genes labeled with double asterisk (**) were identified in the previous study conducted by Pol et al., using 16HBE14o- cells incubated with *A*. *fumigatus* conidia for 6 hours. Proteins significant under BH-FDR < 0.30 are in bold.
(DOCX)

S3 Table Trans-Epithelial Electrical Resistance (TEER) values of 12 ALI cultures exposed to *A*. *fumigatus* conidia for 6 hours. (DOCX)

S4 Table RNA transcript data for 770 genes profiled using NanoString’s Immune Profiling Panel. (XLSX)
