# Supplementary figures and images for: Transcriptomic and proteomic host response to Aspergillus fumigatus conidia in an air-liquid interface model of human bronchial epithelium
Source: PLoS One. 2018 Dec 27;13(12):e0209652. doi: 10.1371/journal.pone.0209652 (PMC6307744; doi:10.1371/journal.pone.0209652)

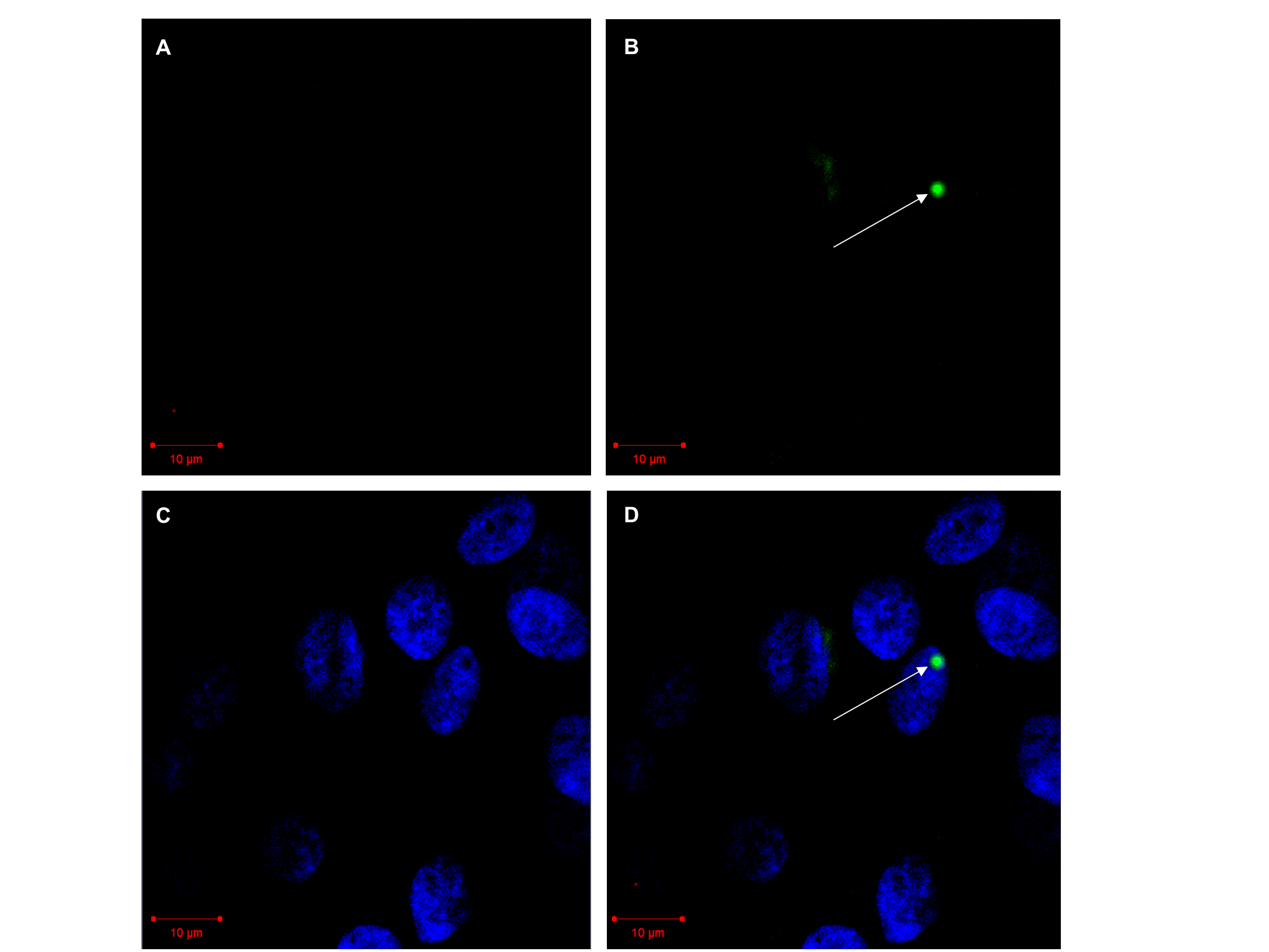

Supplement: S1 Fig — GFP-expressing A. fumigatus conidia and primary HBECs grown in ALI were co-incubated for 6 hours, fixed and stained with DAPI to label cell nuclei, and a monoclonal anti-A. fumigatus antibody was used to label extracellular conidia, before visualization using confocal microscopy. One representative field is shown in the following channels: (A) wavelength 594nm for anti-A. fumigatus antibody (red); (B) wavelength 495nm for GFP (green); (C) wavelength 405nm for DAPI (blue); (D) merged GFP, anti-A. fumigatus antibody and DAPI image. Conidia not labeled by the anti-A. fumigatus antibody and only visible in the green but not the red channel were considered to be internalized by ALI cultures of primary HBECs. Field of view is 1912x1912 pixels, and scale bar is 10 μm. (TIF) [file pone.0209652.s001.tif]

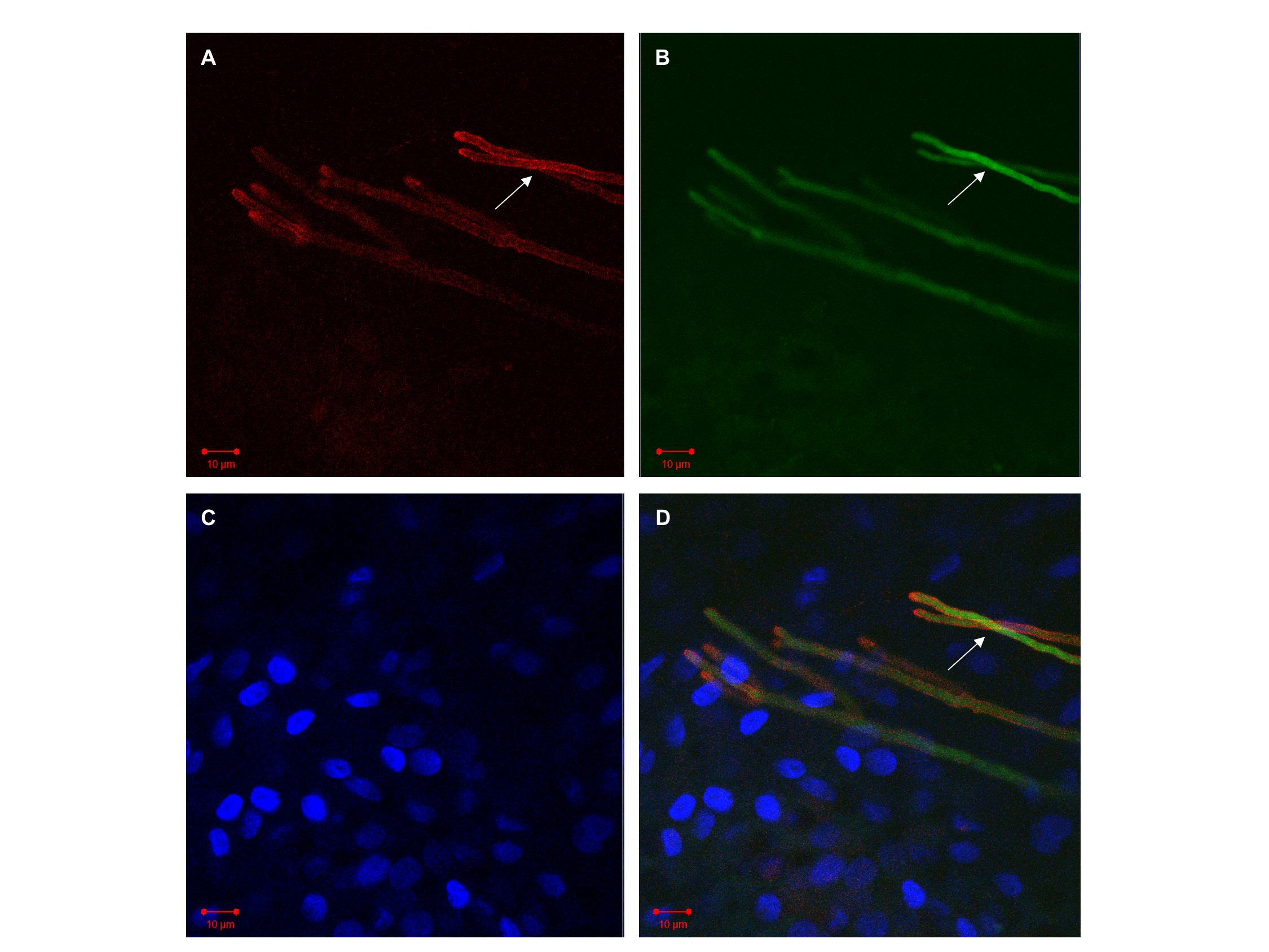

Supplement: S2 Fig — GFP-expressing A. fumigatus conidia and primary HBECs grown in ALI were co-incubated for 24 hours and processed as described in Fig 2 legend. (A) wavelength 594nm for anti-A. fumigatus antibody (red); (B) wavelength 495nm for GFP (green); (C) wavelength 405nm for DAPI (blue); (D) merged GFP, anti-A. fumigatus antibody and DAPI image. Hyphae (white arrows) germinated from the bound conidia is shown. Field of view is 512x512 pixels with a zoom of 2x, and the scale bar is 10 μm. (TIF) [file pone.0209652.s002.tif]
