# Supplementary material for: Transcriptomic and proteomic host response to Aspergillus fumigatus conidia in an air-liquid interface model of human bronchial epithelium
Source: PLoS One. 2018 Dec 27;13(12):e0209652. doi: 10.1371/journal.pone.0209652 (PMC6307744; doi:10.1371/journal.pone.0209652)
Supplement: S1 Table — mRNA transcripts significant under BH-FDR < 0.30 are in bold. (DOCX) [file pone.0209652.s003.docx]

|  | **Gene** | **logFC** | **Ave Expr** | **t** | **P.Value** | **adj.P.Val** | **B** |
| --- | --- | --- | --- | --- | --- | --- | --- |
| 1 | **MAF** | **-0.44359** | **6.82573** | **-4.06966** | **0.00172** | **0.29984** | **-2.89905** |
| 2 | **LCN2** | **-0.45200** | **14.59534** | **-3.60385** | **0.00391** | **0.29984** | **-3.13435** |
| 3 | **TFE3** | **0.49446** | **5.38698** | **3.36555** | **0.00606** | **0.29984** | **-3.26973** |
| 4 | **SELPLG** | **0.83025** | **5.16622** | **3.33491** | **0.00640** | **0.29984** | **-3.28689** |
| 5 | **BST2** | **0.50296** | **6.95952** | **3.27159** | **0.00718** | **0.29984** | **-3.32270** |
| 6 | **IDO1** | **-1.01796** | **9.72366** | **-3.27033** | **0.00719** | **0.29984** | **-3.32341** |
| 7 | **CFB** | **-0.44695** | **12.14702** | **-3.26346** | **0.00721** | **0.29984** | **-3.32324** |
| 8 | **ERCC3** | **0.38350** | **7.30922** | **3.24726** | **0.00742** | **0.29984** | **-3.33221** |
| 9 | **IL6R** | **0.65195** | **6.27056** | **3.21873** | **0.00790** | **0.29984** | **-3.35293** |
| 10 | **COG7** | **0.43241** | **8.67394** | **3.17591** | **0.00845** | **0.29984** | **-3.37385** |
| 11 | **CCL15** | **0.90273** | **4.91337** | **3.13488** | **0.00919** | **0.29984** | **-3.40152** |
| 12 | **IFNL1** | **0.64642** | **5.33877** | **3.07619** | **0.01022** | **0.30564** | **-3.43596** |
| 13 | IL5RA | 0.81702 | 7.15057 | 2.99326 | 0.01187 | 0.31053 | -3.48521 |
| 14 | CASP8 | 0.27555 | 7.29066 | 2.90551 | 0.01381 | 0.31053 | -3.53484 |
| 15 | FADD | 0.36815 | 5.39405 | 2.88802 | 0.01425 | 0.31053 | -3.54551 |
| 16 | SAA1 | -0.61278 | 14.60114 | -2.86647 | 0.01494 | 0.31053 | -3.56178 |
| 17 | PIN1 | 0.42598 | 8.26600 | 2.85131 | 0.01535 | 0.31053 | -3.57102 |
| 18 | FCF1 | 0.34814 | 9.84935 | 2.81085 | 0.01640 | 0.31053 | -3.59289 |
| 19 | MFGE8 | -0.53263 | 9.88091 | -2.81371 | 0.01643 | 0.31053 | -3.59404 |
| 20 | RRAD | 0.59030 | 8.93488 | 2.77177 | 0.01773 | 0.31481 | -3.61985 |
| 21 | NFATC2 | 0.42765 | 5.57471 | 2.73922 | 0.01881 | 0.31481 | -3.63998 |
| 22 | CTSS | 0.50947 | 11.02793 | 2.72516 | 0.01929 | 0.31481 | -3.64869 |
| 23 | FEZ1 | -0.38363 | 8.06035 | -2.64154 | 0.02244 | 0.31657 | -3.70081 |
| 24 | CD164 | 0.25005 | 11.37882 | 2.63469 | 0.02258 | 0.31657 | -3.70272 |
| 25 | CD44 | -0.42655 | 10.84923 | -2.61059 | 0.02373 | 0.31657 | -3.72021 |
| 26 | CXCL6 | -0.67177 | 11.64939 | -2.59126 | 0.02457 | 0.31657 | -3.73235 |
| 27 | IL2RG | -0.45634 | 5.23796 | -2.56257 | 0.02587 | 0.31657 | -3.75042 |
| 28 | SPA17 | 0.71513 | 11.11601 | 2.55330 | 0.02631 | 0.31657 | -3.75626 |
| 29 | ALAS1 | 0.27812 | 8.55835 | 2.53975 | 0.02681 | 0.31657 | -3.76270 |
| 30 | LCK | 0.66207 | 5.21615 | 2.53469 | 0.02721 | 0.31657 | -3.76801 |
| 31 | C3 | -0.46736 | 12.90939 | -2.53203 | 0.02734 | 0.31657 | -3.76969 |
| 32 | ANXA1 | 0.40989 | 14.60464 | 2.51434 | 0.02822 | 0.31659 | -3.78087 |
| 33 | CXCL5 | -1.14653 | 9.02873 | -2.44410 | 0.03201 | 0.33151 | -3.82541 |
| 34 | CD24 | 0.29284 | 12.72601 | 2.43897 | 0.03214 | 0.33151 | -3.82683 |
| 35 | ATF1 | 0.30605 | 7.87936 | 2.43592 | 0.03232 | 0.33151 | -3.82877 |
| 36 | PLA2G6 | 0.39003 | 5.74706 | 2.36019 | 0.03719 | 0.36685 | -3.87882 |
| 37 | SYK | 0.26675 | 7.32229 | 2.34840 | 0.03781 | 0.36685 | -3.88473 |
| 38 | BCL2L1 | 0.26137 | 11.10980 | 2.30450 | 0.04089 | 0.37758 | -3.91284 |
| 39 | TNFRSF11A | -0.40192 | 6.54937 | -2.30520 | 0.04102 | 0.37758 | -3.91391 |
| 40 | CASP3 | 0.26494 | 9.92874 | 2.24540 | 0.04542 | 0.38075 | -3.95073 |
| 41 | MERTK | 0.26278 | 5.68801 | 2.20550 | 0.04875 | 0.38075 | -3.97630 |
