# Supplementary material for: Transcriptomic and proteomic host response to Aspergillus fumigatus conidia in an air-liquid interface model of human bronchial epithelium
Source: PLoS One. 2018 Dec 27;13(12):e0209652. doi: 10.1371/journal.pone.0209652 (PMC6307744; doi:10.1371/journal.pone.0209652)
Supplement: S2 Table — 8 genes labeled with double asterisk (**) were identified in the previous study conducted by Pol et al., using 16HBE14o- cells incubated with A. fumigatus conidia for 6 hours. Proteins significant under BH-FDR < 0.30 are in bold. (DOCX) [file pone.0209652.s004.docx]

|  | **Gene** | **logFC** | | **AveExpr** | **t** | **P.Value** | **adj.P.Val** | **B** |
| --- | --- | --- | --- | --- | --- | --- | --- | --- |
| 1 | **CALR** | **5.72296** | | **5.72296** | **29.47107** | **0.0000005** | **0.00089** | **3.70815** |
| 2 | **NUCB2** | **2.76004** | | **2.76004** | **10.35694** | **0.00011** | **0.06737** | **1.61191** |
| 3 | **SET** | **2.69194** | | **2.69194** | **10.28771** | **0.00011** | **0.06737** | **1.58944** |
| 4 | **MATR3** | **1.73914** | | **1.73914** | **9.19187** | **0.00020** | **0.07522** | **1.19608** |
| 5 | **TPM3;DKFZp686J1372** | **1.74663** | | **1.74663** | **9.08705** | **0.00021** | **0.07522** | **1.15440** |
| 6 | **CBX5** | **1.31134** | | **1.31134** | **7.05672** | **0.00072** | **0.19998** | **0.17182** |
| 7 | **EEA1** | **1.21880** | | **1.21880** | **6.59847** | **0.00100** | **0.19998** | **-0.10522** |
| 8 | **EIF4B** | **1.34810** | | **1.34810** | **6.59087** | **0.00100** | **0.19998** | **-0.11002** |
| 9 | **TMEM205** | **-0.87489** | | **-0.87489** | **-5.81094** | **0.00099** | **0.19998** | **-0.17469** |
| 10 | **RDX** | **1.17865** | | **1.17865** | **6.42933** | **0.00113** | **0.20248** | **-0.21363** |
| 11 | **ST13;ST13P5;ST13P4** | **1.32255** | | **1.32255** | **6.20186** | **0.00134** | **0.20541** | **-0.36498** |
| 12 | **NME1** | **1.27855** | | **1.27855** | **6.16663** | **0.00137** | **0.20541** | **-0.38900** |
| 13 | **HMGB3** | **1.35247** | | **1.35247** | **5.75821** | **0.00189** | **0.26094** | **-0.67951** |
| 14 | **EIF3J** | **1.05370** | | **1.05370** | **5.62692** | **0.00210** | **0.26108** | **-0.77777** |
| 15 | **LSM8** | **1.37488** | | **1.37488** | **5.47232** | **0.00239** | **0.26108** | **-0.89666** |
| 16 | **INS;INS-IGF2** | **1.06686** | | **1.06686** | **4.92386** | **0.00236** | **0.26108** | **-1.04124** |
| 17 | **ALB** | **-1.61075** | | **-1.61075** | **-4.87969** | **0.00248** | **0.26108** | **-1.08759** |
| 18 | **CFAP58** | **0.94581** | | **0.94581** | **5.16217** | **0.00311** | **0.27517** | **-1.14591** |
| 19 | **RSPH4A** | **0.94347** | | **0.94347** | **5.15343** | **0.00314** | **0.27517** | **-1.15315** |
| 20 | **HDGF** | **0.94134** | | **0.94134** | **5.13432** | **0.00319** | **0.27517** | **-1.16901** |
| 21 | **UBXN1** | **0.93814** | | **0.93814** | **5.07921** | **0.00335** | **0.27517** | **-1.21510** |
| 22 | **ALYREF** | **1.04981** | | **1.04981** | **5.07020** | **0.00338** | **0.27517** | **-1.22267** |
| 23 | LRRFIP1** | | 0.90230 | 0.90230 | 4.71811 | 0.00464 | 0.35025 | -1.52912 |
| 24 | KTN1 | | 1.29127 | 1.29127 | 4.70766 | 0.00469 | 0.35025 | -1.53852 |
| 25 | HDAC1 | | 0.85978 | 0.85978 | 4.61618 | 0.00511 | 0.35219 | -1.62166 |
| 26 | RRBP1 | | 1.02929 | 1.02929 | 4.51269 | 0.00563 | 0.37412 | -1.71742 |
| 27 | KRT1 | | -0.79086 | -0.79086 | -4.23219 | 0.00502 | 0.35219 | -1.80396 |
| 28 | SBDS | | -0.90827 | -0.90827 | -4.41308 | 0.00620 | 0.38953 | -1.81133 |
| 29 | SNRPE | | 0.81285 | 0.81285 | 4.39667 | 0.00630 | 0.38953 | -1.82697 |
| 30 | ATP6V0D1 | | -0.79455 | -0.79455 | -4.23908 | 0.00736 | 0.41216 | -1.97952 |
| 31 | AP2M1 | | -0.56892 | -0.56892 | -3.97634 | 0.00674 | 0.40306 | -2.10628 |
| 32 | CHMP2A | | -0.81106 | -0.81106 | -4.08437 | 0.00859 | 0.42930 | -2.13357 |
| 33 | EIF2S2 | | 0.84864 | 0.84864 | 4.05977 | 0.00881 | 0.42930 | -2.15845 |
| 34 | API5 | | -0.60699 | -0.60699 | -3.92988 | 0.00712 | 0.41205 | -2.16235 |
| 35 | ODF2 | | 0.74095 | 0.74095 | 3.95798 | 0.00978 | 0.43992 | -2.26258 |
| 36 | ETF1 | | -0.92092 | -0.92092 | -3.82712 | 0.00805 | 0.42489 | -2.28760 |
| 37 | HSPE1 | | -0.65222 | -0.65222 | -3.82659 | 0.00806 | 0.42489 | -2.28826 |
| 38 | YBX1;YBX3;YBX2 | | 0.75167 | 0.75167 | 3.88876 | 0.01052 | 0.44549 | -2.33444 |
| 39 | PPP1CA | | -0.55032 | -0.55032 | -3.74789 | 0.00886 | 0.42930 | -2.38537 |
| 40 | RPL13 | | -0.80619 | -0.80619 | -3.71773 | 0.00919 | 0.43359 | -2.42285 |
| 41 | PLGRKT | | 0.85480 | 0.85480 | 3.78172 | 0.01177 | 0.46411 | -2.44728 |
| 42 | SERPINB4;SERPINB3 | | -0.54233 | -0.54233 | -3.66380 | 0.00981 | 0.43992 | -2.49022 |
| 43 | SEC11A | | 0.82671 | 0.82671 | 3.73292 | 0.01240 | 0.46411 | -2.49941 |
| 44 | NASP | | 0.74792 | 0.74792 | 3.72871 | 0.01246 | 0.46411 | -2.50392 |
| 45 | KRT2 | | -1.23232 | -1.23232 | -3.60867 | 0.01050 | 0.44549 | -2.55957 |
| 46 | EIF4E | | -0.74466 | -0.74466 | -3.65987 | 0.01342 | 0.46411 | -2.57823 |
| 47 | NDUFAF2 | | 0.83641 | 0.83641 | 3.65533 | 0.01348 | 0.46411 | -2.58317 |
| 48 | DHX15 | | -0.50393 | -0.50393 | -3.58093 | 0.01087 | 0.44549 | -2.59465 |
| 49 | ISYNA1 | | -0.66913 | -0.66913 | -3.64376 | 0.01365 | 0.46411 | -2.59574 |
| 50 | ESYT2 | | 0.78023 | 0.78023 | 3.57616 | 0.01093 | 0.44549 | -2.60069 |
| 51 | PRKCSH | | 1.06681 | 1.06681 | 3.63507 | 0.01378 | 0.46411 | -2.60521 |
| 52 | CRIP2** | | 0.59493 | 0.59493 | 3.48924 | 0.01218 | 0.46411 | -2.71142 |
| 53 | IPO4 | | -0.76666 | -0.76666 | -3.49703 | 0.01603 | 0.46834 | -2.75736 |
| 54 | S100P | | 0.64847 | 0.64847 | 3.49635 | 0.01605 | 0.46834 | -2.75811 |
| 55 | C12orf10 | | 0.65603 | 0.65603 | 3.49140 | 0.01614 | 0.46834 | -2.76364 |
| 56 | STX12 | | -0.78664 | -0.78664 | -3.43421 | 0.01720 | 0.46834 | -2.82772 |
| 57 | CKMT1A;CKMT1B | | -0.46602 | -0.46602 | -3.39159 | 0.01377 | 0.46411 | -2.83719 |
| 58 | CPT1A | | -0.85461 | -0.85461 | -3.42450 | 0.01738 | 0.46834 | -2.83865 |
| 59 | RBM47 | | -0.65321 | -0.65321 | -3.41848 | 0.01750 | 0.46834 | -2.84544 |
| 60 | HEXA | | -0.53532 | -0.53532 | -3.38001 | 0.01398 | 0.46411 | -2.85220 |
| 61 | SNRPA | | -0.49907 | -0.49907 | -3.35343 | 0.01446 | 0.46547 | -2.88672 |
| 62 | HNRNPC;HNRNPCL1 | | 1.58017 | 1.58017 | 3.34906 | 0.01454 | 0.46547 | -2.89240 |
| 63 | TP53BP1 | | 0.69088 | 0.69088 | 3.36815 | 0.01852 | 0.47436 | -2.90244 |
| 64 | ARFGAP2 | | -0.69670 | -0.69670 | -3.35522 | 0.01879 | 0.47455 | -2.91716 |
| 65 | RELA | | 0.64402 | 0.64402 | 3.27974 | 0.01588 | 0.46834 | -2.98294 |
| 66 | BUB3 | | -0.44757 | -0.44757 | -3.25582 | 0.01638 | 0.46834 | -3.01434 |
| 67 | TMEM14C | | 0.59921 | 0.59921 | 3.26378 | 0.02085 | 0.49983 | -3.02202 |
| 68 | POLR2C | | -0.63170 | -0.63170 | -3.26139 | 0.02091 | 0.49983 | -3.02478 |
| 69 | RPL7A | | -0.57434 | -0.57434 | -3.24605 | 0.01659 | 0.46834 | -3.02717 |
| 70 | RPL13A | | -0.60359 | -0.60359 | -3.23669 | 0.01679 | 0.46834 | -3.03949 |
| 71 | RPL28 | | -0.65822 | -0.65822 | -3.21726 | 0.01721 | 0.46834 | -3.06511 |
| 72 | ACADSB | | -0.63380 | -0.63380 | -3.19468 | 0.02257 | 0.51236 | -3.10220 |
| 73 | PGRMC1 | | 0.53587 | 0.53587 | 3.17861 | 0.01810 | 0.47300 | -3.11620 |
| 74 | RPS20 | | 0.51895 | 0.51895 | 3.17413 | 0.01820 | 0.47300 | -3.12212 |
| 75 | COG5 | | -0.57998 | -0.57998 | -3.15890 | 0.02353 | 0.52736 | -3.14402 |
| 76 | STMND1 | | 0.71323 | 0.71323 | 3.13267 | 0.02426 | 0.52948 | -3.17481 |
| 77 | RAB10 | | 0.66297 | 0.66297 | 3.12472 | 0.01941 | 0.48340 | -3.18775 |
| 78 | DNAJC10 | | -0.58361 | -0.58361 | -3.11837 | 0.02467 | 0.52948 | -3.19165 |
| 79 | ANK3 | | -1.33082 | -1.33082 | -3.10577 | 0.02503 | 0.52948 | -3.20651 |
| 80 | TXNDC12 | | 0.54822 | 0.54822 | 3.10075 | 0.02003 | 0.49194 | -3.21969 |
| 81 | CFAP36 | | 0.58540 | 0.58540 | 3.04006 | 0.02704 | 0.53112 | -3.28441 |
| 82 | GBE1 | | -0.62675 | -0.62675 | -3.04820 | 0.02146 | 0.50626 | -3.28997 |
| 83 | KIF5B | | 0.56744 | 0.56744 | 3.01499 | 0.02785 | 0.53112 | -3.31431 |
| 84 | LMO7 | | 0.58382 | 0.58382 | 3.01145 | 0.02797 | 0.53112 | -3.31854 |
| 85 | RPL3 | | -0.51794 | -0.51794 | -3.02467 | 0.02213 | 0.51192 | -3.32154 |
| 86 | GSTT1 | | -0.43706 | -0.43706 | -3.02004 | 0.02227 | 0.51192 | -3.32776 |
| 87 | ENKUR | | 0.62207 | 0.62207 | 2.95959 | 0.02975 | 0.53112 | -3.38072 |
| 88 | H2AFY2** | | -0.59469 | -0.59469 | -2.94411 | 0.03030 | 0.53112 | -3.39934 |
| 89 | UGGT1 | | -0.67482 | -0.67482 | -2.94379 | 0.03031 | 0.53112 | -3.39974 |
| 90 | MANF | | -0.75803 | -0.75803 | -2.94025 | 0.03044 | 0.53112 | -3.40400 |
| 91 | SOD1 | | -0.57894 | -0.57894 | -2.96076 | 0.02409 | 0.52948 | -3.40761 |
| 92 | APOO | | 0.53770 | 0.53770 | 2.92769 | 0.03090 | 0.53112 | -3.41916 |
| 93 | RPLP0;RPLP0P6 | | -0.40564 | -0.40564 | -2.92979 | 0.02510 | 0.52948 | -3.44947 |
| 94 | TBCB | | -0.75197 | -0.75197 | -2.90010 | 0.03194 | 0.53112 | -3.45252 |
| 95 | PSMA4 | | 0.70297 | 0.70297 | 2.89110 | 0.03229 | 0.53112 | -3.46344 |
| 96 | HSP90AA1** | | 1.33988 | 1.33988 | 2.91700 | 0.02553 | 0.53112 | -3.46678 |
| 97 | RSPH3 | | 0.57347 | 0.57347 | 2.86304 | 0.03340 | 0.53351 | -3.49751 |
| 98 | SNX12 | | -0.68226 | -0.68226 | -2.85744 | 0.03362 | 0.53351 | -3.50432 |
| 99 | UBQLN1 | | 0.41236 | 0.41236 | 2.88319 | 0.02671 | 0.53112 | -3.51264 |
| 100 | ATP5L | | -0.50211 | -0.50211 | -2.88314 | 0.02671 | 0.53112 | -3.51271 |
| 101 | MCU | | -0.52275 | -0.52275 | -2.84919 | 0.03396 | 0.53413 | -3.51437 |
| 102 | FTL** | | -0.67758 | -0.67758 | -2.85177 | 0.02786 | 0.53112 | -3.55535 |
| 103 | ASPH | | -0.40332 | -0.40332 | -2.84071 | 0.02828 | 0.53112 | -3.57042 |
| 104 | RPL27 | | -0.53245 | -0.53245 | -2.80189 | 0.03596 | 0.54158 | -3.57215 |
| 105 | PREP | | -0.42289 | -0.42289 | -2.83877 | 0.02835 | 0.53112 | -3.57305 |
| 106 | RPS8 | | -0.53285 | -0.53285 | -2.83489 | 0.02850 | 0.53112 | -3.57834 |
| 107 | BCCIP | | 0.51170 | 0.51170 | 2.77610 | 0.03711 | 0.54158 | -3.60379 |
| 108 | EIF4A3 | | -0.42784 | -0.42784 | -2.79937 | 0.02990 | 0.53112 | -3.62679 |
| 109 | ABRACL | | 0.51896 | 0.51896 | 2.75658 | 0.03801 | 0.54158 | -3.62777 |
| 110 | LDHA** | | -0.51040 | -0.51040 | -2.79600 | 0.03003 | 0.53112 | -3.63139 |
| 111 | DCTN1;DKFZp686E0752 | | 0.50775 | 0.50775 | 2.75009 | 0.03831 | 0.54158 | -3.63577 |
| 112 | CYFIP1;CYFIP2 | | -0.44521 | -0.44521 | -2.79154 | 0.03021 | 0.53112 | -3.63749 |
| 113 | MYO6 | | 0.52172 | 0.52172 | 2.73328 | 0.03910 | 0.54352 | -3.65648 |
| 114 | GLG1 | | -0.37932 | -0.37932 | -2.76502 | 0.03131 | 0.53112 | -3.67375 |
| 115 | CDK5 | | -0.57636 | -0.57636 | -2.75814 | 0.03161 | 0.53112 | -3.68315 |
| 116 | PFKP | | -0.42249 | -0.42249 | -2.75309 | 0.03182 | 0.53112 | -3.69008 |
| 117 | SF1 | | -0.75727 | -0.75727 | -2.74767 | 0.03206 | 0.53112 | -3.69748 |
| 118 | EIF1;EIF1B | | 0.49427 | 0.49427 | 2.69085 | 0.04120 | 0.55333 | -3.70892 |
| 119 | S100A11 | | 0.78714 | 0.78714 | 2.71967 | 0.03330 | 0.53351 | -3.73586 |
| 120 | RPL17 | | -0.77599 | -0.77599 | -2.71671 | 0.03343 | 0.53351 | -3.73992 |
| 121 | TEKT2 | | 0.83154 | 0.83154 | 2.66355 | 0.04261 | 0.55358 | -3.74277 |
| 122 | LMNA | | 0.36964 | 0.36964 | 2.69458 | 0.03446 | 0.53721 | -3.77030 |
| 123 | FGFR1OP | | 0.48869 | 0.48869 | 2.62366 | 0.04476 | 0.56923 | -3.79239 |
| 124 | APEH | | -0.38335 | -0.38335 | -2.66966 | 0.03565 | 0.54158 | -3.80454 |
| 125 | SF3B5 | | 0.72859 | 0.72859 | 2.61077 | 0.04549 | 0.57002 | -3.80845 |
| 126 | KRAS** | | 0.39786 | 0.39786 | 2.66225 | 0.03601 | 0.54158 | -3.81473 |
| 127 | TMED7;TICAM2 | | -0.41772 | -0.41772 | -2.66214 | 0.03601 | 0.54158 | -3.81489 |
| 128 | HDHD3 | | -0.65187 | -0.65187 | -2.60003 | 0.04610 | 0.57002 | -3.82186 |
| 129 | SNRNP200 | | -0.53801 | -0.53801 | -2.59338 | 0.04648 | 0.57019 | -3.83016 |
| 130 | PIR | | 0.47667 | 0.47667 | 2.58879 | 0.04675 | 0.57019 | -3.83590 |
| 131 | MYH9 | | 0.78867 | 0.78867 | 2.64148 | 0.03704 | 0.54158 | -3.84331 |
| 132 | RPS18 | | -0.39185 | -0.39185 | -2.64125 | 0.03706 | 0.54158 | -3.84363 |
| 133 | CAST | | 1.03540 | 1.03540 | 2.62633 | 0.03782 | 0.54158 | -3.86417 |
| 134 | IGFBP3 | | -0.40502 | -0.40502 | -2.62031 | 0.03813 | 0.54158 | -3.87246 |
| 135 | MAP2K3 | | -0.67652 | -0.67652 | -2.55684 | 0.04865 | 0.57765 | -3.87586 |
| 136 | UQCRB | | -0.49097 | -0.49097 | -2.61596 | 0.03836 | 0.54158 | -3.87846 |
| 137 | SRP14 | | 0.54208 | 0.54208 | 2.54755 | 0.04922 | 0.58055 | -3.88750 |
| 138 | ALDH1A1 | | -0.51660 | -0.51660 | -2.60703 | 0.03883 | 0.54352 | -3.89076 |
| 139 | HIST1H4A | | -0.41764 | -0.41764 | -2.57641 | 0.04050 | 0.55333 | -3.93300 |
| 140 | RPS6 | | -0.49713 | -0.49713 | -2.55926 | 0.04147 | 0.55333 | -3.95666 |
| 141 | TMED2 | | 0.44778 | 0.44778 | 2.55511 | 0.04170 | 0.55333 | -3.96239 |
| 142 | F11R | | -0.50950 | -0.50950 | -2.55407 | 0.04176 | 0.55333 | -3.96383 |
| 143 | G6PD | | -0.42977 | -0.42977 | -2.55081 | 0.04195 | 0.55333 | -3.96833 |
| 144 | DHRS7 | | -0.39571 | -0.39571 | -2.55048 | 0.04197 | 0.55333 | -3.96879 |
| 145 | RPS11 | | -0.36969 | -0.36969 | -2.54488 | 0.04229 | 0.55354 | -3.97652 |
| 146 | RECQL | | -0.36164 | -0.36164 | -2.52953 | 0.04320 | 0.55723 | -3.99771 |
| 147 | VWA5A | | -0.45330 | -0.45330 | -2.51851 | 0.04386 | 0.56172 | -4.01294 |
| 148 | RPS5 | | -0.43004 | -0.43004 | -2.49502 | 0.04531 | 0.57002 | -4.04541 |
| 149 | LRPAP1 | | 0.44064 | 0.44064 | 2.48498 | 0.04594 | 0.57002 | -4.05929 |
| 150 | AARS | | -0.35524 | -0.35524 | -2.46276 | 0.04737 | 0.57393 | -4.09003 |
| 151 | GMPPA | | -0.34083 | -0.34083 | -2.45492 | 0.04789 | 0.57631 | -4.10087 |
| 152 | CHMP1A | | 0.64099 | 0.64099 | 2.44598 | 0.04849 | 0.57765 | -4.11323 |
| 153 | CMAS | | -0.33708 | -0.33708 | -2.42971 | 0.04960 | 0.58120 | -4.13574 |
