# Supplementary material for: Transcriptomic and proteomic host response to Aspergillus fumigatus conidia in an air-liquid interface model of human bronchial epithelium
Source: PLoS One. 2018 Dec 27;13(12):e0209652. doi: 10.1371/journal.pone.0209652 (PMC6307744; doi:10.1371/journal.pone.0209652)
Supplement: S3 Table — (DOCX) [file pone.0209652.s005.docx]

| **12-well plate format** | | **24-well plate format** | |
| --- | --- | --- | --- |
| Sample | TEER value  (Ohms) | Sample | TEER value (Ohms) |
| Control | 360 | Control | 1030 |
| Control | 440 | Control | 1029 |
| Control | 130 | Control | 1022 |
| Infected | 405 | Infected | 1268 |
| Infected | 380 | Infected | 1056 |
| Infected | 115 | Infected | 1197 |
